# Recent Advances in Patterning Strategies for Full-Color Perovskite Light-Emitting Diodes

**DOI:** 10.1007/s40820-023-01254-8

**Published:** 2023-12-07

**Authors:** Gwang Heon Lee, Kiwook Kim, Yunho Kim, Jiwoong Yang, Moon Kee Choi

**Affiliations:** 1 Graduate School of Semiconductor Materials and Devices Engineering, Center for Future Semiconductor Technology (FUST), Ulsan National Institute of Science and Technology (UNIST), Ulsan, 44919 Republic of Korea; 2 Department of Energy Science and Engineering, Daegu Gyeongbuk Institute of Science and Technology (DGIST), Daegu, 42988 Republic of Korea; 3 Energy Science and Engineering Research Center, Daegu Gyeongbuk Institute of Science and Technology (DGIST), Daegu, 42988 Republic of Korea; 4 Department of Materials Science and Engineering, Ulsan National Institute of Science and Technology (UNIST), Ulsan, 44919 Republic of Korea; 5 Center for Nanoparticle Research, Institute for Basic Science (IBS), Seoul, 08826 Republic of Korea

**Keywords:** Perovskite, Light-emitting diode, Full-color display, High-resolution patterning, Electroluminescence

## Abstract

Metal halide perovskites have emerged as promising light-emitting materials for next-generation displays owing to their remarkable material characteristics including broad color tunability, pure color emission with remarkably narrow bandwidths, high quantum yield, and solution processability. Despite recent advances have pushed the luminance efficiency of monochromic perovskite light-emitting diodes (PeLEDs) to their theoretical limits, their current fabrication using the spin-coating process poses limitations for fabrication of full-color displays. To integrate PeLEDs into full-color display panels, it is crucial to pattern red–green–blue (RGB) perovskite pixels, while mitigating issues such as cross-contamination and reductions in luminous efficiency. Herein, we present state-of-the-art patterning technologies for the development of full-color PeLEDs. First, we highlight recent advances in the development of efficient PeLEDs. Second, we discuss various patterning techniques of MPHs (i.e., photolithography, inkjet printing, electron beam lithography and laser-assisted lithography, electrohydrodynamic jet printing, thermal evaporation, and transfer printing) for fabrication of RGB pixelated displays. These patterning techniques can be classified into two distinct approaches: in situ crystallization patterning using perovskite precursors and patterning of colloidal perovskite nanocrystals. This review highlights advancements and limitations in patterning techniques for PeLEDs, paving the way for integrating PeLEDs into full-color panels.

## Highlights

- This article reviews the recent progress in the patterning techniques of metal halide perovskites for full-color displays.
- Patterning techniques of perovskites are subdivided into in situ crystallization and patterning of colloidal perovskite nanocrystals, including photolithography, inkjet printing, thermal evaporation, laser ablation, transfer printing, and so on.
- The strength and weakness of each patterning methods are discussed in detail from the viewpoint of their applications in full-color displays.

## Graphical abstract

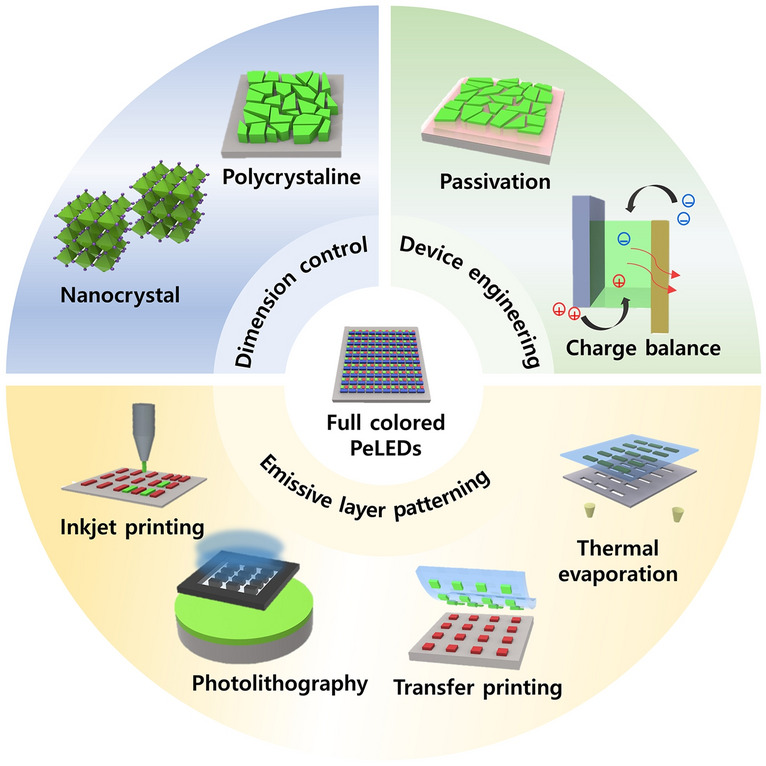

## Introduction

In the era of Metaverse, the demand for advanced displays, particularly head-mounted and near-eye displays for augmented reality (AR) and virtual reality (VR) applications, has grown exponentially to deliver unparalleled visual experiences and to meet the ever-increasing expectations of consumers across diverse industries [1, 2]. The development of high-resolution displays with vivid and accurate colors has become paramount in order to enhance image quality, readability, and user immersion [3, 4]. To meet the general resolution requirement of approximately 60 pixels per degree for human eyes [5], AR/VR head-mounted displays and smart glasses necessitate high pixel density. For instance, compared to larger conventional displays like smartphones (e.g., Galaxy S23 with 425 pixels-per-inch (PPI), Samsung Electronics) and TVs (e.g., 8K television with 104 PPI), near-eye displays such as 2-inch smart glasses may require exceptionally high pixel density, reaching up to 3,000 PPI. Consequently, the development of high-definition patterning technologies becomes indispensable in creating sharp and well-defined pixel structures.

Metal halide perovskites (MHPs) have emerged as promising emitters for light-emitting diodes (LEDs) due to their advantageous properties such as narrow full-width-at-half-maximum (FWHM) [6, 7], spectral tunability over all the visible region [8, 9], high photoluminescence quantum yields (PLQYs) [10] and defect tolerance [11]. Particularly, the sharp emission width (15–20 nm) with wide color gamut and superior contrast ratio of perovskite light-emitting diodes (PeLEDs) offer vivid and realistic displays, which are suitable for VR and AR displays compared to the conventional liquid crystal displays and organic light-emitting diodes (OLEDs). Since the development of the first room-temperature-operating electroluminescent (EL) device based on perovskites in 2014 [12], significant efforts have been dedicated to improving the device performance, resulting in the remarkable external quantum efficiency (EQE) of PeLEDs (25.8% [13], 28.9% [14], and 18% [15] for red, green, and blue PeLEDs, respectively), which is close to the theoretical limits. However, despite the remarkable results achieved thus far, it is crucial to note that these advancements have primarily been demonstrated in monochromatic PeLEDs fabricated via the spin-coating process [16–18]. Therefore, there are several challenges involved in the commercial-scale production of high-definition full-color displays such as development of patterning techniques for red–green–blue (RGB) subpixels that can effectively operate within EL devices.

Table 1 presents a range of printing techniques, including inkjet printing, photolithography, nanoimprinting, and transfer printing, which have been proposed for patterning MHPs in optoelectronic devices. To achieve high-resolution RGB patterns using MHPs, it is crucial to develop a printing process that takes into account the vulnerability of MHPs to solvents, oxygen, moisture, and heat exposure. In addition, achieving successful fabrication of full-color EL devices mandates a meticulous approach toward integrating patterned HMPs with charge transport/blocking layers, buffer layers, and electrodes. Conditions such as solvents, light, heat, and pressure used in the patterning process may affect the underneath layers (i.e., charge transport layers (CTLs) and electrodes) and pre-patterned different colored MHPs during the fabrication of PeLEDs, causing cross-contamination or reducing device efficiency. For example, utilizing solvent orthogonality is essential to maintain intact surface morphology and optical/electrical characteristics of underlying CTLs without elution. Detailed strategies for the efficient patterned PeLED implementation will be discussed in the following chapters.

**Table 1:** Comparison of perovskite patterning method

Patterning method	Factor for damage device	Multi-color	Advantage	Disadvantage	References
Photo lithography	UV, PR	○	Accurate alignment	UV, PR damage to emissive layer	[19–21]
Inkjet printing	Organic additive	○	Accurate alignment Orthogonality	Organic additive Pattern size limitation Coffee ring effect	[22–25]
E-jet printing	Organic additive	○	Small pattern size	Low throughput	[26–29]
Transfer printing	**–**	○	Orthogonality Eco-friend	Low throughput	[30, 31]
Thermal evaporation	–	○	Large-area pattering	Pattern size limitation	[32, 33]
Laser ablation	Heat, laser	×	Small pattern size Accurate patterning	Low throughput Damage to bottom layer	[34, 35]
E-beam lithography	E-beam	×	Small pattern size	Low throughput Damage to bottom layer	[36]
laser Scribing	Heat, light	×	Accurate patterning	Low throughput Damage to bottom layer	[37, 38]
X-ray lithography	X-ray	×	Small pattern size	Low throughput Damage to bottom layer	[39]

**Fig. 1:**
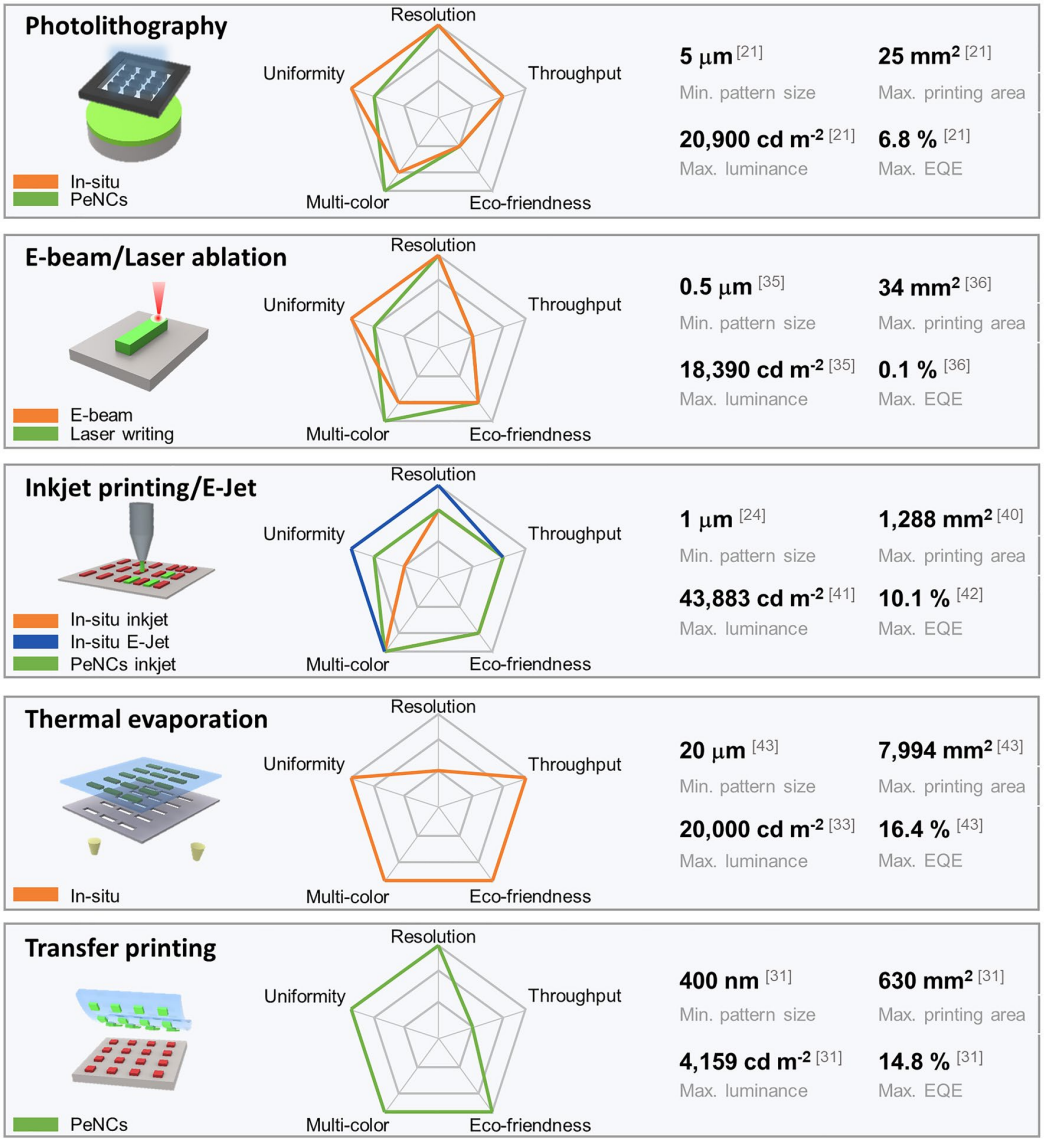
The schematic and radar plot of representative MHP patterning techniques. The radar plot illustrates the evaluation of different MHP patterning techniques based on five key factors: uniformity, resolution, throughput, eco-friendness, and multi-color. The uniformity factor indicates the consistency in morphology, shape, and thickness of each patterned pixels. The eco-friendness factor assesses the energy consumption per unit patterned pixel and the potential generation of chemical contaminants during the patterning process

In this review, we aim to provide a comprehensive account of recent progresses in perovskite patterning methods, specifically concentrating on the pixelization of the perovskite layer using non-destructive techniques and their applications in EL devices. Initially, we examine the crucial parameters and strategies for fabricating high efficiency PeLEDs, particularly in terms of the emissive layer and device engineering. This review classifies the patterning process of perovskite film into two distinct approaches, in-situ crystallization patterning and perovskite nanocrystals (PeNCs) patterning. In the first section, we introduce in situ crystallization patterning techniques using perovskite precursor including photolithography, beam and laser lithography, inkjet, electrohydrodynamic jet (E-jet), and thermal evaporation. In the second section, we discuss the PeNC patterning techniques such as photolithography, inkjet, and transfer printing by using pre-synthesized PeNCs. Figure 1, Tables 2 and 3 provide a comprehensive comparative summary illustrating the distinct characteristics of diverse patterning methods. Lastly, we offer prospective on the achievement of high-resolution perovskite patterns and practical applications of PeLEDs for full-color displays.

**Table 2:** The summary of characteristics of PeLEDs with in-situ crystallization patterning

Fabrication method	Materials	Min pattern size (μm)	Max printing area (mm^2^)	Max EQE [%]	Max luminance (cd m^−2^)	References
Inkjet printing	CsPbBr_3_	125	~ 700	2.12 (G)	2,500	[44]
Inkjet printing	CsPbBr_3_	30	1,288	9.0 (G)	3,640 (G)	[40]
kjet printing	CsPbX_3_	50	10	3.5 (R) 3.4 (G) 1.0 (B)	267 (R) 956 (G) 146 (B)	[22]
Inkjet printing	MAPbBr_3_	250	–	0.8 (G)	10,227 (G)	[23]
Inkjet printing	CsPbBr_3_	50	10	10.1 (G)	12,882 (G)	[42]
Inkjet printing	FAPbBr_3_	45	400	4.5 (G)	12,738 (G)	[45]
Laser lithography	CsPbBr_3_	0.5	–	CE = 1.9 cd A^−1^ (G)	18,390 (G)	[35]
Thermal evaporation	CsPbBr_3_/ Cs_4_PbBr_6_	100	4,020	8.0 (G)	~ 20,000 (G)	[33]
Thermal evaporation	CsPbBr_3_-TPPO ^a)^	20	7,994	16.4 (G)	~ 10,000 (G)	[43]
Photo lithography	(PEABr)_x_CsPbBr_3_	20	~ 1.1	1.2 (G)	13,043 (G)	[20]
E-beam lithography	MAPbX_3_ (X = I, Br, Cl)	20	~ 34	~ 0.1 (G)	~ 1800 (G)	[36]

a) TPPO: triphenylphosphine oxide

**Table 3:** The summary of characteristics of PeLEDs with PeNCs patterning

Fabrication method	Materials	Min pattern size (μm)	Max printing area (mm^2^)	Max EQE (%)	Max luminance (cd m^−2^)	References
Photolithography	CsPbX_3_	5	25	6.8 (G)	20,900 (G)	[21]
Inkjet printing	FA_0.3_Cs_0.7_PbBr_3_	50	225	2.8 (G)	1,233 (G)	[46]
Inkjet printing	MAPbX_3_	1	–	CE = 6.7 cd A^−1^ (G)	9,700 (G)	[24]
Inkjet printing	CsPbX_3_	~ 70	1232	5.5 (R) 8.5 (G) 0.8 (B)	455 (R) 43,883 (G) 151 (B)	[41]
Inkjet printing	CsPbX_3_	~ 30	1024	0.83 (R) 0.42 (G) 0.05 (B)	272 (R) 379 (G) 22.8 (B)	[25]
Transfer printing	CsPbX_3_	10	225	10.5 (R) 6.7 (B)	~ 680 (R) ~ 350 (B)	[30]
Transfer printing	CsPbX_3_	0.4	~ 630	15.3 (R) 14.8 (G) 2.5 (B)	~ 300 (R) 4,159 (G) ~ 200 (B)	[31]

## Perovskite Light-Emitting Diodes

**Fig. 2:**
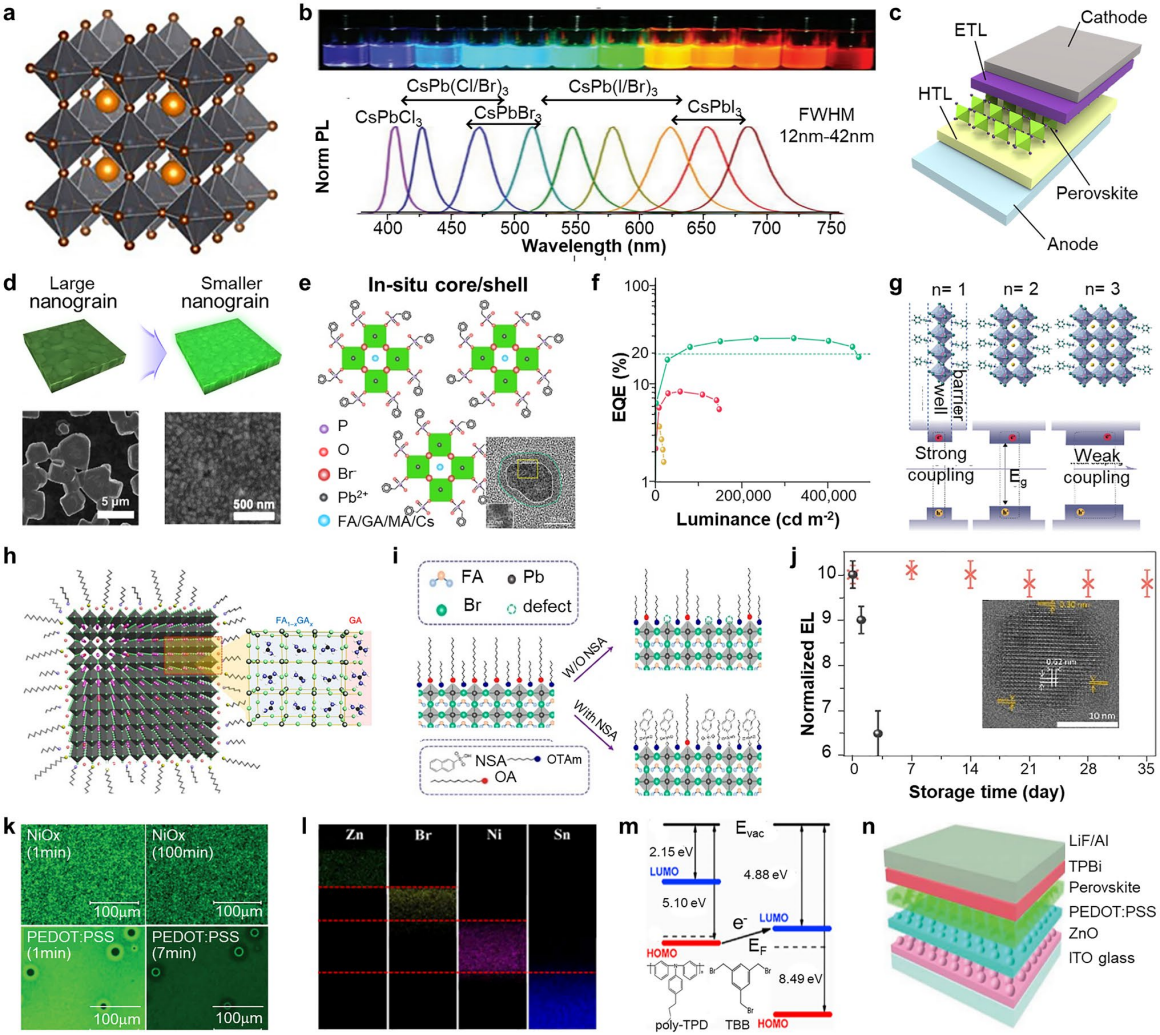
Strategy for highly efficient PeLEDs. **a** Schematic of 3D cubic MHP crystal structure of ABX_3_. Reproduced with permission [47]. Copyright 2019 American Chemical Society. **b** Representative perovskite colloidal solution and photoluminescence (PL) spectra of MHP. Reproduced with permission [48]. Copyright 2015 American Chemical Society. **c**) Schematic of PeLED device structure. **d** Schematics (top) and corresponding scanning electron microscope (SEM) images before (bottom left) and after (bottom right) the nanocrystal pinning process. Reproduced with permission [60]. Copyright 2015 American Association for the Advancement of Science (AAAS). **e** Schematic of in-situ core/shell perovskite with BPA treatment. The inset shows transmission electron microscope (TEM) image of in-situ core/shell perovskite. **f** EQE versus luminance of in-situ core/shell PeLED. Reproduced with permission [14]. Copyright 2022 Springer Nature. **g** Schematic crystal structures of quasi-2D perovskites with varying n values and their corresponding electronic properties influenced by quantum- and dielectric-confinement effects. Reproduced with permission [61]. Copyright 2021 Springer Nature. **h** Schematic of GA doped FAPbBr_3_ PeNCs. Reproduced with permission [69]. Copyright 2021 Springer Nature. **i** Schematic illustration of ligand exchange process of PeNCs with NSA. Reproduced with permission [73]. Copyright 2021 American Chemical Society. **j** Time-related storage stability of normal CsPbI_3_ and Pb capped CsPbI_3_-0.1 PeLEDs under ambient environment. The inset shows TEM image of CsPbI_3_ with 0.3-nm-thick Pb shell. Reproduced with permission [81]. Copyright 2018 American Chemical Society. **k** EL images of quasi-2D PeLEDs with NiO_x_ (top) and PEDOT:PSS (bottom) as HTL at various operation time. Reproduced with permission [87]. Copyright 2018, Wiley–VCH. **l** EDS elemental mapping image of ZnO/CsPbBr_3_/NiO/ITO structure. Reproduced with permission [89]. Copyright 2018 American Chemical Society. **m** Schematic representation of p-type doping mechanism involving electron transfer from Poly-TPD to TBB. The inset shows chemical structure of each molecule. Reproduced with permission [89]. Copyright 2020 Elsevier. **n** Schematic illustration of PeLED device structure with moth-eye nanostructured layer. Reproduced with permission [95]. Copyright 2019, Wiley–VCH

MHP, which stands for metal halide perovskite, is commonly represented by the chemical formula ABX_3_ (Fig. 2a) [47]. In this formula, A represents an organic or inorganic cation, such as formamidinium (FA^+^), methylammonium (MA^+^), and Cs^+^. The B site is occupied by elements such as Pb^2+^, Sn^2+^, while the X site is occupied by halide anions, including I^−^, Br^−^, and Cl^−^. The optical properties and stability of MHP can be finely adjusted by altering the elemental composition. For example, the color of MHP can be finely tuned from near infrared to violet region (Fig. 2b) [48]. Due to the unique defect tolerance nature of MHPs, MHPs show extremely narrow FWHM (< 15 nm) and high color purity compared to organic and quantum dot emitters [49, 50]. Moreover, MHP exhibits a direct bandgap, enabling highly efficient light emission and absorption. This property renders them well-suited for optoelectronic applications, including LEDs, photodetectors, solar cells, and lasers.

Figure 2c shows the typical device structure of PeLEDs, where perovskite emitter is sandwiched between hole transport layer (HTL) and electron transport layer (ETL). The EQE is a widely employed metric for quantifying the efficiency of converting incoming electrons into photons within the PeLEDs. It is expressed using Eq. 1:

1 $$EQE={\eta }_{I}\times {\eta }_{R}\times {\eta }_{O}$$

where $\eta$_I_ is the charge injection efficiency associated with the charge imbalance within the emissive layer, $\eta$_R_ is the radiative recombination efficiency influenced by trap density and excitonic binding energy, and ${\eta }_{O}$ is the outcoupling efficiency related to unhindered photon emission from the device. Current studies on highly efficient PeLEDs primarily focus on increase those factors with various method. $\eta$_R_ can be increased through dimension size control of MHPs, enabling increase of exciton binding energy. In addition, passivating trap sites on the perovskite surface effectively reduce the nonradiative recombination [51, 52]. $\eta$_I_ can be enhanced through device engineering techniques, including the implementation of blocking layers, mobility adjustment, and interface passivation [53, 54].

Before delving into the discussion on patterning techniques for full-color pixelated PeLEDs, it is crucial to provide a brief overview of the strategies employed in highly efficient PeLEDs fabricated by typical spin-coating procedure. By understanding these key strategies, we can better comprehend the reasoning behind the subsequent discussion on patterning techniques.

### Polycrystalline MHPs

The polycrystalline MHP based PeLEDs were widely studied in the early stages of PeLED research due to their simple film formation procedure through a fast and simple spin-coating process [55]. However, polycrystalline MHP films initially exhibited low efficiency in terms of their optoelectrical properties compared to organic emitters. This was primarily attributed to their small exciton binding energy, which led to enhanced dissociation of electron–hole and carriers captured in trap states, ultimately mitigating radiative recombination [56]. Furthermore, the rapid crystallization of MHP can give rise to inadequate surface morphology and incomplete coverage, resulting to the formation of pinholes that affect optical/electrical performance of PeLEDs [57].

Adopting a smaller grain size presents a potential solution to enhance the device performance through effectively increased exciton binding energy and enhanced radiative recombination [58, 59]. For example, Cho et al. demonstrated highly efficient PeLEDs (current efficiency = 42.9 cd A^−1^, EQE = 8.5%) using the nanocrystal pinning process, in which PeNCs were immobilized through fast crystallization induced by volatile anti-solvents (Fig. 2d) [60]. The volatile solvents (i.e., chloroform) swiftly wash out the low volatile precursor solvents [i.e., *N*,*N*-dimethylformamide (DMF) or dimethyl sulfoxide (DMSO)] during the spin-coating process, leading to small and uniform MAPbBr_3_ grains (~ 100 nm) by reducing evaporation time. In contrast, without nanocrystal pinning, the formation of rough micrometer-sized MAPbBr_3_ grains with pinholes occurs, which can lead to a poor interface with CTLs within PeLEDs.

To enhance the optoelectrical performance, it is necessary to passivate these defect sites during device fabrication. Recently, Lee group exhibited defect-passivated core/shell PeNCs achieved through in-situ reaction of benzyl phosphonic acid (BPA) with 3D polycrystalline perovskite films [14]. During the reaction, the polycrystalline MHP was fragmented into nanograin structures. Simultaneously, exposed bromide vacancies on the surface of MHP are efficiently passivated by BPA, through the formation of Pb−O−P covalent bonds. This process significantly reduces the trap density of the emissive layer, while maintaining excellent carrier transport characteristics of 3D perovskites. Based on the core/shell PeNCs, highly efficient and stable PeLEDs were demonstrated with an EQE of 28.9%, a maximum luminance of 420,000 cd m^−2^, and a stability of 30,000 h at 100 cd m^−2^ (Fig. 2e).

Another approach to mitigate the issue of polycrystallinity in perovskite structures involves the formation of quasi-2D perovskite films, which exhibit growth along the < 100 > crystallographic direction with sliced by bulk organic cation between the 3D structure (Fig. 2f) [61]. It is represented as chemical formula A’_2_A_n−1_B_n_X_3n+1_, where n and A’ represent the number of MPH layers and a bulk organic cation (i.e., monoammonium or diammonium cations), respectively. The mixed-dimensional perovskite film shows a unique self-assembled multiple quantum-well structure, which imparts a large exciton binding energy attributed to the quantum confinement effect. Photocarriers within the film tend to transfer from regions with smaller bandgaps (corresponding to lower values of n) to regions with larger bandgaps (higher n values), resulting in carrier accumulation. In the case of mixed 2D﻿–3D perovskite structures, efficient energy transfer occurs within a remarkably short time span of 1 ps [62]. This rapid energy transfer facilitates effective trap filling and contributes to achieving highly efficient PeLEDs [63–65].

### Nanocrystal MHPs

The ligand-passivated colloidal PeNCs are synthesized through hot injection method and ligand-assisted reprecipitation. The PeNCs show great potential as emitting materials for highly efficient LEDs due to their strong spatial confinement of electron–hole pairs. Additionally, beyond the Bohr radius of PeNCs, the size distribution does not impact the emission wavelength. This characteristic results in a narrow FWHM of 15–20 nm for green emission. However, the high surface-to-volume ratio of PeNCs and their low defect formation energy makes them susceptible to the formation of surface defect sites, such as halide vacancies and Pb vacancies. These vacancies can negatively impact the optical performance of PeLEDs by causing nonradiative recombination [66].

To mitigate these issues, doping PeNCs emerges as a promising strategy [67, 68]. For instance, Kim et al. exhibited lattice stabilization by doping zero-dipole guanidinium (GA) cations in FAPbBr_3_ PeNCs (Fig. 2g) [69]. The additional amino group in GA can easily forms hydrogen bonding on the PeNCs, providing lattice-stabilizing effect. In addition, 1,3,5-tris(bromomethyl)-2,4,6-triethylbenzene is employed to passivate the halide vacancies on the surface of the PeNCs. As a result, the PeLEDs achieved a current efficiency of 108 cd A^−1^ and an EQE of 23.4%.

During the synthetic procedure, long chain molecules like oleic acid and oleylamine are commonly employed as surface ligands for PeNCs to facilitate surface passivation. However, it is worth noting that these ligands can also present a challenge by impeding efficient carrier injection into PeNCs within PeLEDs. Accumulated carriers on the surface of PeNCs can reduce device performance through Auger recombination and thermal induced defect formation caused by nonradiative recombination [70, 71]. Hence, ligand engineering involving the manipulation of ligand density and types becomes crucial for the fabrication of high efficiency PeLEDs. Ligand exchange with conjugated or aromatic functional groups can effectively improve charge injection into the PeNCs [72, 73]. For example, Zhao et al. demonstrated highly efficient PeLEDs by developing PeNCs capped with 2-naphthalenesulfonic acid (NSA). The sulfonic group in NSA provides stronger interactions with FAPbBr_3_ PeNCs compared to the pristine oleic acid ligands (Fig. 2h) [73]. Ligand exchange with NSA significantly promotes charge carrier injection into the PeNCs, resulting in high optical properties in PeLEDs, including an EQE of 19.2% and a current efficiency of 85.4 cd A^−1^.

The most promising approach for surface passivation of PeNCs is the use of core/shell structures, similar to metal chalcogenide quantum dots (e.g., CdSe/ZnS or InP/ZnSe). Various materials can be employed for surface coverage of PeNCs, including polymers [74, 75], oxides [76], perovskites [77, 78], and chalcogenides [78]. The selection of shell materials in core/shell PeNCs should be carefully considered based on the intended application. For LED applications, the bandgap alignment known as "type 1" is preferred, where the conduction band minimum (CBM) of the core materials is lower than the CBM of the shell materials, and the valance band maximum (VBM) of the core materials is higher than the VBM of the shell materials [79]. Furthermore, to achieve homogeneous growth of the shell materials on the surface of PeNCs, the lattice mismatch between the core and shell materials is less than 15% [80]. Zhang et al. demonstrated the CsPbI_3_ PeNCs capped with PbS shell material. PbS exhibits low lattice mismatch (< 5%) with CsPbI_3_, enabling successful epitaxial growth [81]. The PbS shell effectively passivates the PeNCs against moisture, temperature, and oxygen. In addition, the PeLEDs fabricated with core/shell PeNCs showed remarkable storage stability compared to PeLEDs with pure CsPbI_3_ PeNCs (Fig. 2i).

### Device Engineering

In the previous section, we discussed recent progress in luminescence and efficiency of PeLEDs by modification of MHPs. Further improvement of device performance can be achieved through the engineering of CTLs including hole transport layer (HTL) and electron transport layer (ETL). The selection of CTLs significantly influences on effective charge transport and charge balance in the emissive layer. In addition, the CTLs can passivate the interface defect sites between CTL and emissive layer and can enhance outcoupling efficiency, resulting in enhanced device performance [82, 83].

Among various HTLs, PEDOT:PSS is the most widely used one for PeLEDs. However, the acidic nature of the PEDOT:PSS can easily lead to the corrosion of the bottom electrode, rendering it vulnerable to long-term operation. In addition, interfacial quenching between perovskite and PEDOT:PSS can directly reduce the light-emitting efficiency. To address these issues, the search for alternative materials to replace PEDOT:PSS has markedly increased [84]. Inorganic HTLs are strong candidates to achieve highly efficient and stable PeLEDs due to their significant advantages, including high chemical stability and higher carrier mobility compared to the organic HTL counterpart [85, 86]. For example, NiO_x_ can be utilized as HTL of quasi-2D PeLEDs because the high conduction band edge position of NiO_x_ can enhance the electron blocking capability [87]. The NiO_x_ based PeLED led to a substantial increase in luminance, rising from 10,600 to 24,100 cd m^−2^, along with a remarkable improvement in EQE from 4.2% to 14.6% compared to PEDOT:PSS-based PeLED. Moreover, the crystallinity of MHP manufactured on NiO_x_ layer was increased, resulting in a significant decrease in interfacial trap density. Consequently, PeLEDs with NiO_x_ exhibited improved stability and marked reduction in susceptibility to ion migration at the interface between the MHP and HTL (Fig. 2k).

In general, inorganic nanoparticles (e.g., ZnO, ZnMgO) have been widely employed as the CTL of quantum dot LEDs owing to their high electron mobility and high electron injection characteristics. However, for the PeLEDs, vapor-evaporated organic layers such as 1,3,5-Tris(1-phenyl-1H-benzimidazol-2-yl)benzene (TPBi) and 1,3,5-Tri(m-pyridin-3-yl phenyl)benzene (TmPyPB) have been commonly used as ETLs because of the vulnerability of MHPs to the solvent and heat exposure. Due to the ionic nature of MHPs, inorganic nanoparticles, typically dispersed in polar solvent, can easily damage the underneath MHP layer [88]. Shi et al. demonstrated all-solution processed PeLEDs by considering the orthogonality of processing solvents [89]. NiO_x_ and ZnO were selected as the HTL and ETL, respectively, owing to their exceptional carrier mobility and thermal stability. Chlorobenzene was employed as the nonpolar solvent to dissolve ZnO nanoparticles, which serves as an orthogonal solvent to prevent damage to the emissive MHP layer. As shown in Fig. 2l, the energy-dispersive X-ray spectroscopy (EDS) elemental mapping analysis of the device's cross section reveals well-defined layers with no intermixing between the HTL, emissive layer, and ETL. As a result, all-inorganic PeLEDs exhibited 6,093 cd m^−2^ and EQE of 3.79%.

Balancing charge injection into emissive layer is one of the key factors for achieving highly efficient PeLEDs. Conventional PEDOT:PSS based HTL shows limited hole injection due to the disparity in HOMO levels compared to that of MHPs, ultimately resulting the imbalance of hole and electron in emissive layer [90]. Hole injection can be enhanced by inserting buffer layer, such as Poly(N,N'-bis-4-butylphenyl-N,N'-bisphenyl)benzidine (poly-TPB), Poly[bis(4-phenyl)(2,4,6-trimethylphenyl)amine (PTAA), Poly(9-vinylcarbazole) (PVK), and Poly(9,9-dioctylfluorene-alt-N-(4-s-butylphenyl)-diphenylamine) (TFB), beneath the emissive layer [12, 91–93]. The decreased energy gap between HTL and MHPs leads to superior device performance through efficient charge injection and transport. For example, 1,3,5-tris(bromomethyl)benzene (TBB) can be incorporated within the PeLED architecture (ITO/PEDOT:PSS/poly-TPD/TBB/FAPbBr_3_ PeNCs/TPBi/LiF/Al) to promote electron delocalization within the poly-TPD by performing as an electron acceptor [6]. This leads to downshift the HOMO level of poly-TPD from –5.10 to –5.42 eV, effectively adjusting band alignment to match the deep HOMO level of perovskites (–5.8 eV) (Fig. 2m). Additionally, the rich bromine group in TBB passivates halide vacancy of MHPs. By optimizing buffer layer using TBB, green PeLED exhibited maximum current efficiency of 77.2 cd A^−1^ and maximum EQE of 20.1%.

Unfortunately, typical $\eta_{{\text{O}}}$ value of PeLEDs is below 30% due to the high refractive index of MHPs (n_perovskite_ > 2.2) compared to that of CTLs (n_organic_ ≈ 1.7–2.0) and substrates (n_substrate_ ≈ 1.5) [17, 94]. This leads the trapping of light caused by total reflection at the interface of MHPs and propagating light through the lateral direction, resulting in decrease of the device performance. Thus, the strategies to increase the light extraction in PeLEDs can effectively enhance the overall device performances. For instance, Shen and co-workers designed bioinspired moth-eye nanostructures in ZnO layer to improve light extraction (Fig. 2j) [95]. Through a simple nanoimprinting process, moth-eye structured ZnO layer was inserted between ITO and PEDOT:PSS, mitigating fresnel reflections at the interface of PeLEDs. This leads to increase of transmittance in the broad range of visible region. As a result, PeLEDs with the patterned ZnO layer showed EQE of 20.3% and current efficiency of 61.9 cd A^−1^ which are 1.5 times higher than those of PeLEDs with flat ZnO layer.

## In-Situ Crystallization Patterning

The terminology ‘in-situ crystallization patterning’ in this paper refers to techniques in which the crystallization process takes place directly at the intended site of pattern formation. A key characteristic of this strategy is that it uses a precursor solution instead of PeNCs in a colloidal solution [96, 97]. The precursor solution is deposited onto a target substrate and undergoes further processes that can promote the crystallization of perovskites. This patterning strategy offers the advantage of enhancing the performance of EL devices by creating a smooth perovskite film with the minimized number of defects [98]. The additional processes such as annealing [99, 100] or the treatment with organic/inorganic halide materials [101–103] may be involved to induce the crystallization. For example, during the annealing process, the evaporation of organic solvent induces a supersaturated state and crystalline transformation by rearrangement of ions [104]. Furthermore, a two-step deposition process has been developed to address the issues of poor uniformity and coverage of one-step spin-coating. In this approach, a solid layer of a single precursor, such as PbI_2_, is first uniformly deposited. Subsequently, through treatment with an organic/inorganic halide solution, molecular intercalation occurs, leading to the formation of uniform perovskite films [105]. In a representative example for MAPbI_3_, the crystal size of the resulting MAPbI_3_ film can be controlled by adjusting the concentration of MAI, which is applied onto the PbI_2_ film [106].

There could be a misconception that in-situ crystallization may have limitations in PeLEDs due to the resulting perovskite film structure composed of large crystals, which have lower exciton energy and, consequently, lower performance. However, recent studies have demonstrated the synthesis of low-dimensional PeNCs with additional functionality through in situ crystallization processes. For instance, incorporating precursor solutions mixed with polymer can induce the formation of PeNCs within the polymer matrix [19, 33]. This approach allows for simultaneous improvements in stability and performance, making it suitable for LED applications. In this chapter, we will discuss the recent developments in patterning technologies that utilize in-situ crystallization techniques and their applications in PeLEDs.

### Photolithography

Photolithography is a well-established patterning technique widely used for the fabrication of fine patterns at the micrometer scale [4]. The process involves selectively exposing specific areas to light (usually, ultraviolet, UV), using a patterned photomask [107, 108]. The process consists of various steps depending on the underlying mechanism of the patterning. Photolithography usually employs light-sensitive chemicals called as photoresists, which undergo chemical reactions upon exposure to light. In the case of positive photoresist, the exposed area is removed because the chemical structures can be altered, making them more soluble in a developer solution. For example, carbon-chain scission reactions of poly(methyl methacrylate) (PMMA) can be mediated by the UV exposure, resulting in depolymerization and subsequent removal of the exposed regions [109]. Contrary, in the case of negative photoresist, the area exposed to light become harden through the polymerization and remains as patterned regions that resist removal during subsequent processing steps [110]. The unique characteristics of photosensitive photoresists allow for selective utilization based on the requirements of the patterning process. Positive and negative photoresists offer distinct advantages depending on the desired outcome and pattern design.

**Fig. 3:**
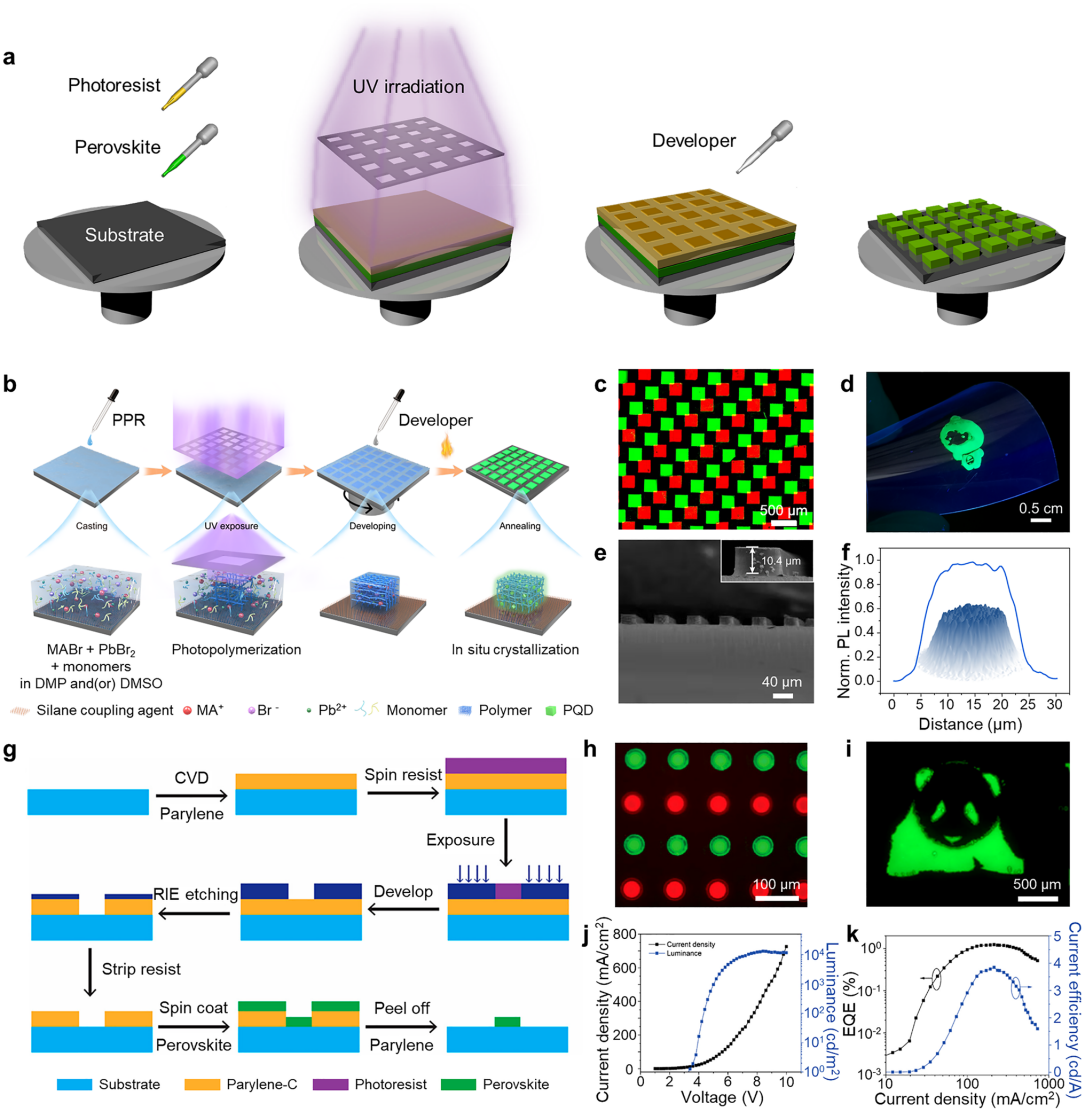
**a** Schematic illustration depicting the top-down photolithography strategy using perovskite precursors and negative photoresist. **b** Schematic representation of the direct in-situ photolithography process utilizing photosensitive PPR. **c** Multi-color (red and green) patterns of squares with the width of 250 μm. **d** Single-color cartoon image. **e** Cross section SEM image showing perovskite stripe patterns polymerized with the same UV exposure time. **f** Radial PL intensity distribution of a circle pixel. Reproduced with permission [19]. Copyright 2022, Springer Nature. **g** Schematic illustration showing the peel-off fabrication process with a parylene-C layer positioned between the photoresist and perovskite layer. **h** Multi-color (red and green) perovskite patterns of circles with a diameter of 50 μm and **i** single-color cartoon image of a panda. **j** *J–V–L* curve and **k** EQE and current efficiency curve measured from PeLEDs using the patterned perovskite layer. Reproduced with permission [20]. Copyright 2020, American Chemical Society

Typically, the photolithography techniques have been implemented for perovskite patterning in two different protocols: top-down and bottom-up photolithography, which differ based on the order of deposition of perovskite materials and photoresist [111]. Figure 3a shows a schematic illustration of the top-down photolithography process using negative photoresist [112]. In a typical procedure, a layer of perovskite is initially deposited onto a substrate, followed by the application of photoresist [110]. The area that is designed to remain as patterns is exposed to UV light, leading to the polymerization of the photoresist in those regions. Subsequently, a developer solution is utilized to remove the unexposed photoresist and the perovskite in the unexposed area is washed away, producing a patterned perovskite film.

It is important to note that perovskite materials, particularly, are highly sensitive to light and various chemicals, and their optical properties can be significantly compromised when exposed to UV light or wet chemicals such as developers [113]. Therefore, to effectively apply the top-down photolithography for perovskite materials, it is crucial to provide comprehensive protection for the perovskite layer during the process. For instance, Harwell et al. proposed a strategy that involves the introduction of a PMMA layer between the photoresist and perovskite layers to minimize potential damage [110]. However, it is challenging to completely prevent damage even with the passivation of perovskite materials.

To overcome this degradation issue, a modified process has been suggested in which all photolithography steps are completed before the crystallization of perovskites. In this modified process, a photosensitive solution, called as perovskite precursor resist (PPR), is prepared by combining multifunctional thiol and ethenyl monomers (trimethylolpropane tris(3-mercaptopropionate) (TTMP) and triallyl isocyanurate (TAIC)), along with MABr and PbBr_2_ precursors in polar aprotic solvents of DMF and DMSO (Fig. 3b) [19]. Subsequently, the prepared solution is deposited on the substrate. Photopolymerization initiated by UV exposure creates patterns of the perovskite precursor solution and the subsequent annealing induces the crystallization of PeNCs within the polymer matrix [114]. Since the UV exposure step occurs prior to perovskite crystallization, photo-induced degradation of the perovskites can be effectively avoided. This method enables the formation of high-resolution patterning with a pixel density of 2,450 PPI and the realization of multi-color patterns, composed of red and green perovskite films (Fig. 3c, d). Additionally, the technique allows for control over the thickness of the perovskite film based on the duration of UV exposure. The resulting film exhibits high experimental reproducibility, with consistent thickness and PL intensity (Fig. 3e). Moreover, the PL intensity distribution on the radial surface of a 20 μm circle pixel appears homogeneous (Fig. 3f). Similarly, Kim et al. developed a technique in which the first step involved patterning the PbBr_2_ layer using photolithography [115]. Subsequently, a dipping process involving CsBr was employed to induce crystallization.

To avoid chemical damages during the application of a developer solution, a bottom-up protocol known as a peel-off method, has been developed [116–118]. This method employs a mold created through photolithography. The perovskite material is deposited onto a substrate with a patterned photoresist layer. Subsequently, the photoresist is removed through a peel-off process, resulting in a patterned perovskite film [119]. Zou et al. introduced a parylene-C layer between the substrate and the photoresist layer (Fig. 3g) [20]. The weak adhesion between the parylene-C mold and the substrate enables complete removal of the mold even after perovskite deposition, leaving only the desired perovskite pattern with the high patterning yield [120]. With this method, a minimum width of 20 μm can be achieved. Furthermore, due to the relatively weak adhesion between paryleneC and perovskite, the patterned perovskite film can be repeatedly covered with paryleneC without causing significant damage to the perovskite. As a result, it becomes possible to protect the patterned perovskite film from UV exposure, enabling successful multi-color patterning (Fig. 3h, i). The PeLEDs using the patterned perovskite layer achieved a maximum luminance of 13,043 cd m^−2^ at 8.4 V and a maximum EQE of 1.24% (Fig. 3j, k) [121]. While photolithography using precursor solutions has not yet achieved high EQE for PeLEDs, recent advancements have shown promising results. In particular, it has demonstrated a noteworthy EQE of up to 9.1% [122], suggesting that photolithography using precursor solutions holds significant potential for PeLED applications.

### E-Beam and Laser-Assisted Lithography

Recently, lithography strategies that utilize electron beam (E-beam) [123–125] or laser irradiation [126, 127] instead of traditional UV irradiation have been explored [128]. E-beam and laser-assisted lithography techniques can offer the capability to selectively irradiate specific areas for pattern formation without a photomask. This selective irradiation is made possible through the use of computer programming, which provides enhanced flexibility and increased degrees of freedom in the patterning process. Consequently, these methods can generate diverse high-resolution patterns with remarkable efficiency and cost-effectiveness [129]. In addition, these techniques can be utilized for the fabrication of substrates and photoresist molds, as well as induce the localized crystallization of perovskite, allowing for precise patterning.

**Fig. 4:**
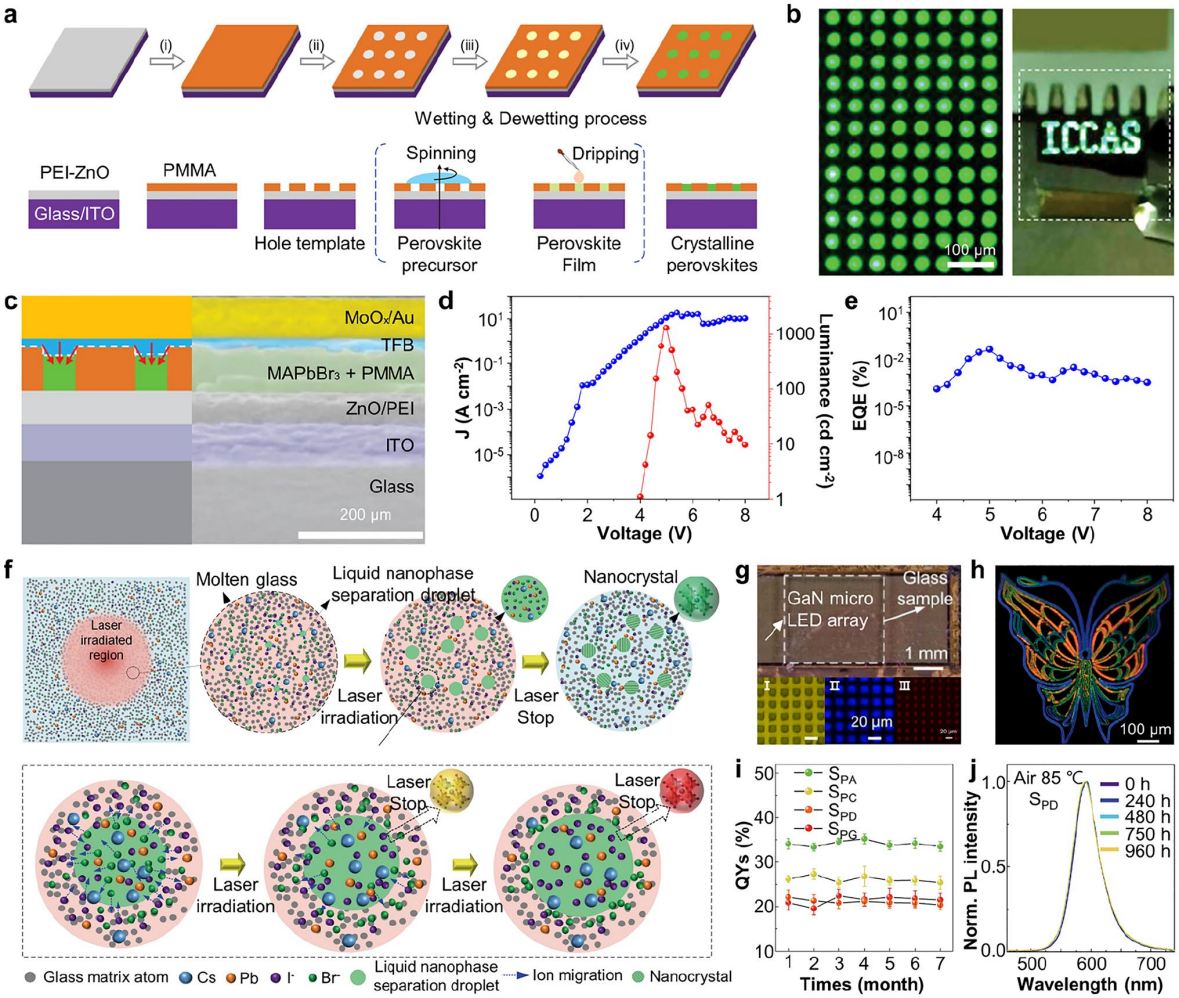
**a** Schematic illustration showing the patterning process utilizing the hole template fabricated through E-beam lithography. **b** EL image of green perovskite circle patterns and “ICCAS” logo photography showing perovskite micro-LED arrays. **c** Schematic illustrating the device structure and cross section SEM image of PeLEDs. **d** *J*–*V*–*L* and **e** EQE curve of the fabricated PeLEDs employing perovskite layers. Reproduced with permission [36]. Copyright 2020, Wiley–VCH. **f** Schematic of the formation of CsPb(Cl_1-x_Br_x_)_3_ nanocrystals induced by ultrafast laser irradiation. **g** Optical microscope image showing the structure of micro-PeLED devices. **h** Multi-color patterned image obtained using ultrafast laser-assisted lithography. **i** Changes in PLQYs of CsPb(Cl_1-x_Br_x_)_3_ nanocrystals exposed to ethanol and** j** PL spectra after heating at 85 °C for 960 h. Reproduced with permission [34]. Copyright 2022, The American Association for the Advancement of Science (AAAS)

The schematic illustration of E-beam assisted lithography is presented in Fig. 4a [36]. In a representative work by Wang et al., a layer of lyophilic ZnO nanoparticles (diameter: ~ 3 nm) covered with polyethyleneimine (PEI) and a lyophobic PMMA layer were deposited, and then a hole template was created using E-beam. When a perovskite precursor solution was spin-coated onto this template, de-wetting occurred on the lyophobic area during spin-coating, causing the migration of perovskite precursors from the lyophobic area to the lyophilic hole [130]. Subsequent crystallization of the perovskite precursor by dripping of anti-solvent resulted in the formation of a patterned perovskite film. This strategy achieved high-resolution, forming patterns with diameters as small as 20 μm (Fig. 4b). Especially, the presence of the unremoved PMMA structure aided in the formation of "current-focusing" architecture in the transporting layer of TFB (Fig. 4c), improving current density (Fig. 4d). The achieved EQE is considerably low (~ 0.1%) for these polycrystalline perovskites (Fig. 4e). One of the strategies that can potentially help to enhance the device performance is utilizing single-crystal perovskites, although previous studies using single-crystalline perovskite patterns fabricated through E-beam lithography have predominantly focused on limited applications, such as solar cells or photodetectors [131–133].

Another method, laser-assisted lithography, utilizes energy transfer to the precursor solution to achieve direct writing of patterns [134]. For example, photon absorption in perovskite through the ultrafast laser irradiation leads to subsequent accumulation of thermal energy [135]. This photon–matter interaction allows control over the crystallinity, size, and shape of PeNCs. Huang et al. demonstrated the reversible in-situ fabrication and decomposition of PeNCs within a transparent oxide glass matrix [136]. CsPbBr_3_ nanocrystals embedded in a matrix exhibited the high stability, and various controlled structure can be formed by adjusting laser characteristics.

Sun et al. further developed a method for growing PeNCs with tunable colors inside oxide glass (40B_2_O_3_-15P_2_O_5_-10Al_2_O_3_-10ZnO-5Na_2_O-5K_2_O-7Cs_2_O-3PbX_2_-5NaX) using a laser-assisted lithography (Fig. 4f) [34]. The energy transfer to highly mobile Cs, Pb, I, and Br ions inside the oxide glass-induced local pressure and temperature increases, facilitating the formation of PeNCs [137]. At this step, the ratio of the halide in complex CsPb(Cl_1−x_Br_x_)_3_ can be controlled by regulating the pulse energy and duration of the laser. This enables the generation of PeNCs in desired colors, ranging from green to red. In addition, the formation of nanocrystals during the cooling process after laser irradiation mitigates damage caused by the laser. The multi-color patterned PeNCs showed efficacy in color filter applications (Fig. 4g, h). The PeNCs embedded inside the glass demonstrated high stability even in harsh environments. Specifically, there was no significant change in PL intensity even after exposure to ethanol for 6 months and the heat treatment at 85 ℃ for 960 h (Fig. 4i, j). The unique structure of PeNCs embedded inside the glass matrix, formed through laser-assisted lithography, offers higher stability compared to perovskite patterns created using other methods, making it promising despite the challenges such as high equipment costs and complicated patterning processes.

### In-Situ Inkjet Patterning

Inkjet printing is a highly efficient patterning technique that enables the direct deposition of functional ink onto a target substrate, eliminating the requirement for patterning masks. This approach offers significant advantages, including the potential for low-cost [138], scalable [139], and customizable fabrication [139] of diverse patterns, making it well-suited for large-scale commercial production [140]. One of the key advantages of inkjet printing is its versatility, as it can be applied to a wide range of substrates, including both rigid and flexible/stretchable materials [141]. The quality of inkjet-printed patterns (i.e., film morphology) is significantly affected by rheological factors, such as solvent viscosity and surface tension of ink. These factors can be quantitatively described by Eq. 2,

2 $$Z= \frac{1}{Oh}= \frac{\sqrt{\gamma \rho \alpha }}{\eta }$$

where *Z* is inverse of Ohnesorge number (Oh), $\upgamma$, $\uprho$, $\mathrm{\alpha }$, and $\eta$ are surface tension, density of fluid, diameter of printing nozzle, and viscosity, respectively [142]. During the printing process, the formation of coffee ring patterns is a common occurrence attributed to the capillary flow [143]. This flow arises as the ink dries and leads to the migration of colloidal particles from the center to the periphery of the printed patterns. The resulting uneven ink morphology can create defect sites and impede the vertical stacking of the CTLs during the fabrication of PeLEDs, thereby leading to nonradiative recombination [143, 144]. Notably, since 2014, inkjet printing has been successfully employed to fabricate MHP patterns for use in optoelectronic devices [145]. Inkjet printing of perovskite offers several benefits, including cost reduction, simple ink preparation, and damage free from UV or processing chemicals [146].

**Fig. 5:**
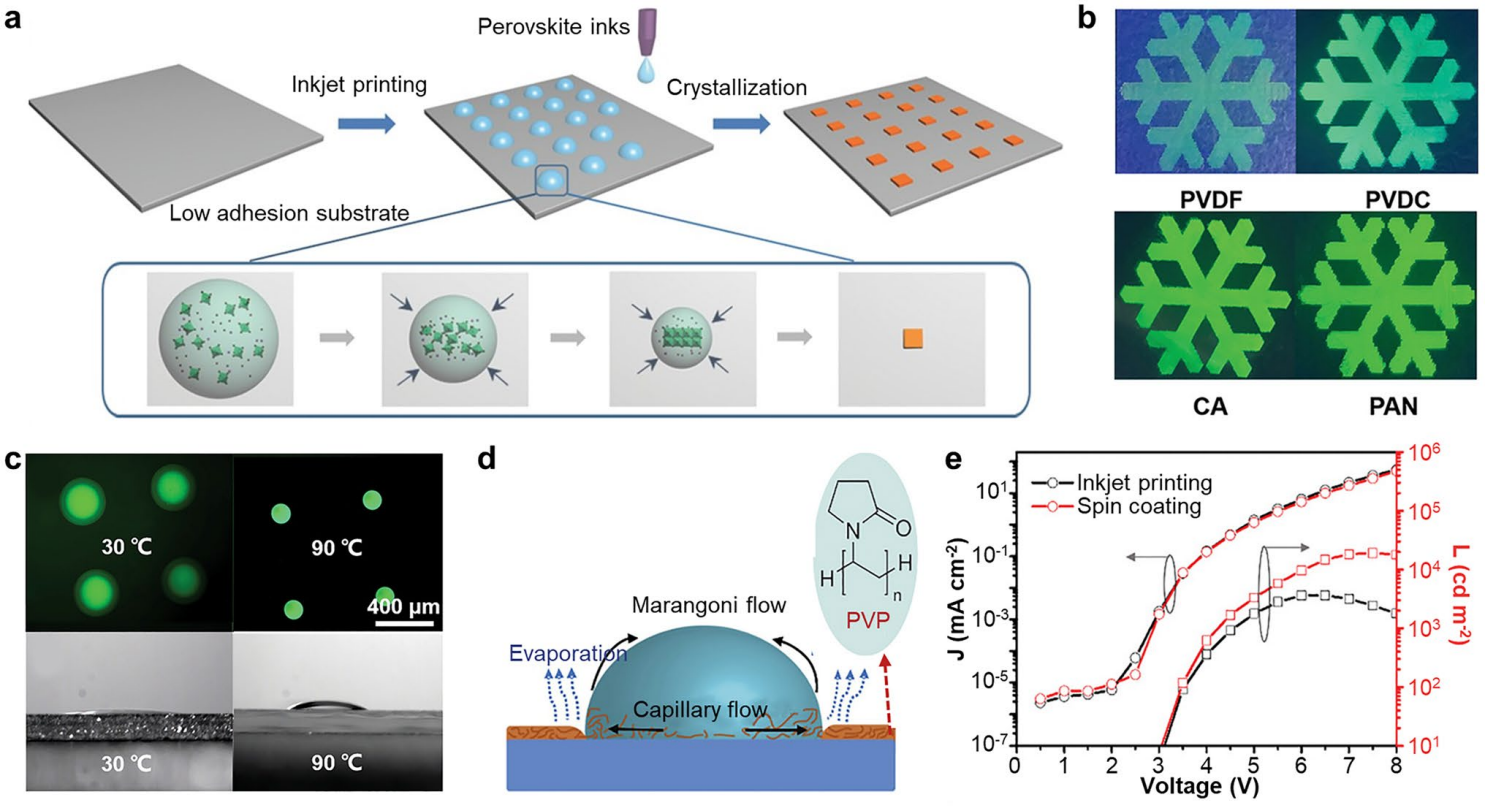
**a** Schematic illustration of the inkjet printing technique using in-situ crystallization of the perovskite precursor. Reproduced with permission [147]. Copyright 2016, Wiley–VCH. **b** PL images of PeNC patterns printed on various polymer substrates. **c** Fluorescence images (top) and cross-sectional contact angle images (bottom) of inkjet-printed dot patterns with different substrate temperatures of 30 and 90 ℃. Reproduced with permission [150]. Copyright 2019, Wiley–VCH. **d** Schematic diagram of the formation principle of coffee ring effect with PVP layer. **e** *J–V–L* curves of PeLEDs fabricated by spin-coating and inkjet printing. Reproduced with permission [40]. Copyright 2021, Wiley–VCH

Recently, inkjet printing of non-crystalline perovskite precursor is widely studied (Fig. 5a). This technique involves ejecting perovskite precursor ink from a nozzle and depositing patterns directly onto the target substrate. As the solvent in the ink evaporates, the precursor ink undergoes in-situ crystallization, resulting in the formation of distinct shape patterns [147]. For the fabrication of PeLEDs, the perovskite precursor needs to print onto the polymeric CTL. When the perovskite precursor solution is deposited onto polymer substrates, the solution induces partial swelling and expansion of the polymer chains, leading to the incorporation of perovskite precursors into the polymer matrix [148]. Subsequently, as the solvent gradually evaporates, the perovskite initiates crystallization, forming PeNCs within the polymer matrix [149]. Specifically, any polymers that are capable of dissolving in the solvent of the perovskite precursor can be utilized as the substrate of the inkjet printing (Fig. 5b) [150]. The pattern size of in-situ crystallized PeNCs is influenced by both the nozzle size and environmental factors, including substrate temperature. Shi et al. exhibited the size of patterned PeNCs was decreased as the substrate temperature increases, because of the change in wettability between precursor ink and polymer matrix (Fig. 5c) [150]. In general, droplet diameter (D) has relationship with contact angle ($\theta$) and volume of droplet, as described in Eq. 3 [151]:

3 $$D=\sqrt[3]{\frac{24V{\mathrm{sin}}^{3}\theta }{2\pi -3\pi \mathrm{cos}\theta + \pi {\mathrm{cos}}^{3}\theta }}(0^\circ < \theta \le 90^\circ )$$

For the PeLED fabrication via inkjet printing, it is crucial to suppress the coffee ring effect to ensure effective layer-by-layer deposition on CTLs. To mitigate this, balancing the capillary flow and Marangoni flow which occur inside the droplet during the evaporation process is necessary. Incorporating interfacial layer between perovskite precursor droplet and HTL can effectively control the surface tension [42, 152]. For example, insertion of hydrophilic polyvinylpyrrolidone (PVP) layer on HTL decreased the surface tension of perovskite precursor ink and enhances Marangoni flow, resulting in thin and uniform ink patterns (Fig. 5d) [40]. The thin insulating PVP layer not only restrained the coffee ring effect but also suppressed leakage current of PeLEDs, resulting in high-performance inkjet-printed red PeLEDs with max EQE of 9.0% and max luminance of 3,640 cd m^−2^ (Fig. 5e).

**Fig. 6:**
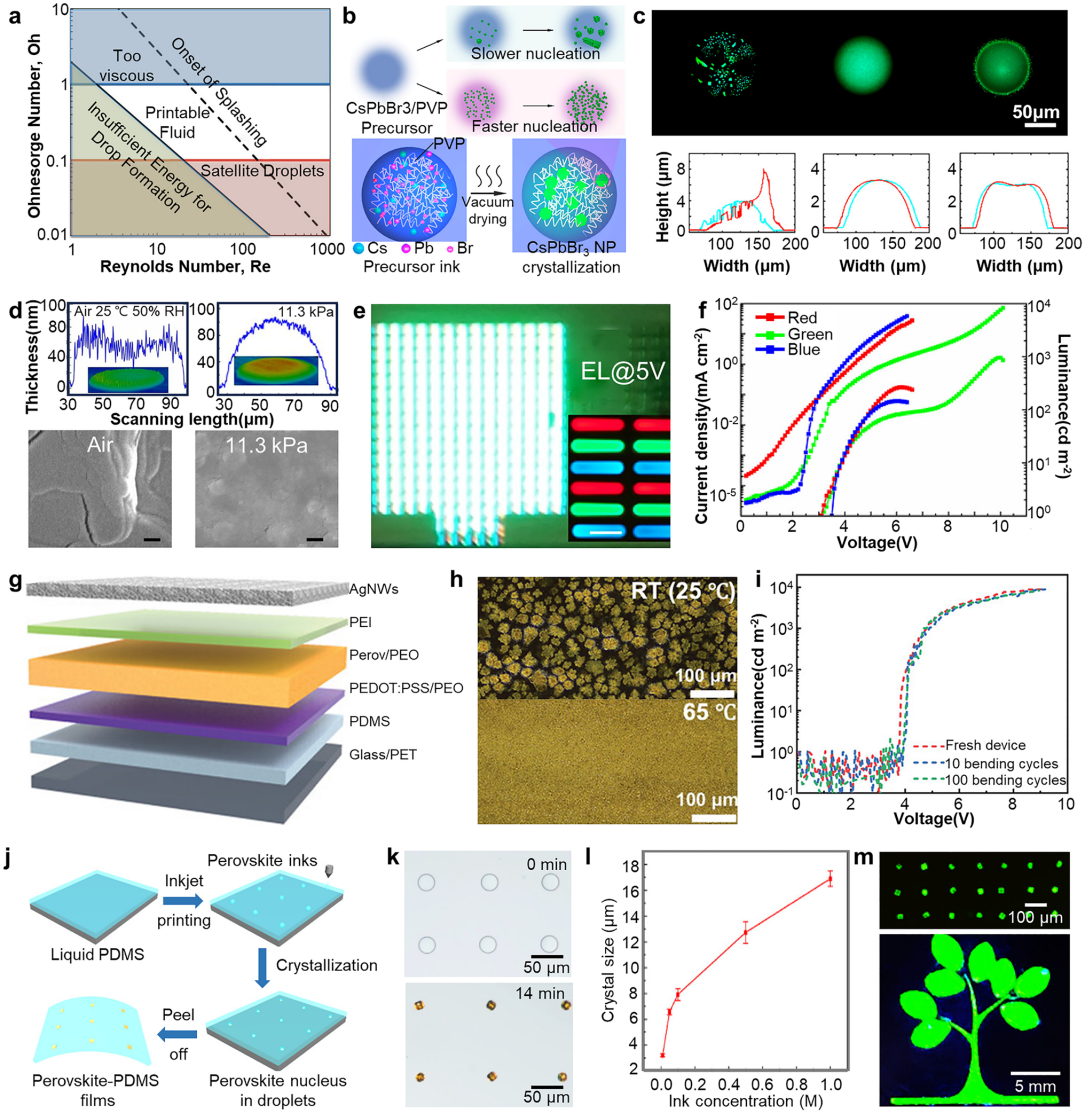
**a** Schematic diagram illustrating operational modes for stable performance in drop-on-demand inkjet printing. Reproduced with permission [155]. Copyright 2011, American Institute of Physics. **b** Schematic showing crystallization of PeNCs with different speed of nucleation (top) and crystallization process of PVP containing precursor ink (bottom). **c** PL images (top) and thickness profile (bottom) of crystallized single dot pattern in ambient condition (left), vacuum condition with substrate temperature of 20 ℃ (middle) and 30 ℃ (right). Reproduced with permission [96]. Copyright 2019, American Chemical Society. **d** Film thickness profile of crystallized single dot pattern (top) and SEM images (bottom) dried at different vacuum level. The insets show 3D morphology of inkjet-printed pattern. Scale bar is 100 nm. **e** EL image of white PeLED at 5 V. The inset shows RGB pixelated patterns. Scale bar is 100 μm. **f** *J–V–L* curves of printed RGB PeLEDs. Reproduced with permission [22]. Copyright 2021, American Chemical Society. **g** Schematic device structure of all-inkjet-printed PeLED. **h** Optical microscope images of composite film printed at different temperature. **i** *L–V* curves of all-inkjet-printed flexible PeLEDs according to bending cycles. Reproduced with permission [23]. Copyright 2021, Wiley–VCH. **j** Schematic illustration of the inkjet printing process for fabrication of single-crystal perovskite-embedded PDMS. **k** Optical images of single-crystal growth according to the growth time. **l** The size of single crystals depending on the ink concentration. **m** Fluorescence images of complicated single-crystal perovskite patterns embedded in PDMS films. Reproduced with permission [159]. Copyright 2020, American Chemical Society

Achieving high-quality perovskite patterns requires excellent ink printability, which relies on smooth jetting of the ink through the nozzle and the formation of stable droplets [45, 153]. The ink's state can be characterized by three parameters: Reynolds number (Re), Weber number (We), and Oh [154]. These parameters describe the balance between inertial, surface, and viscous forces, considering rheological properties such as ink viscosity, density, and surface tension, as well as printing characteristics like nozzle diameter. The stable operational state of ink in inkjet printing can be determined by the relationship between Re, which represents the ratio of inertial force to viscous force, and Oh, which represents the ratio of viscous force to the square root of inertial force multiplied by surface forces (Fig. 6a) [155]. Liu et al. enhanced the crystallinity of inkjet-printed perovskite patterns by incorporating PVP into the precursor ink, resulting in increased viscosity and reduced outward capillary flow (Fig. 6b) [156]. The C=O unit of PVP forms a back-bonding interaction with Pb on the surface of the PeNCs by high adsorption energy between O–Pb, confining the NCs within the PVP matrix [157]. The postprocessing conditions, such as substrate temperature, was critical parameter for the stable printing (Fig. 6c). Under ambient conditions, slow evaporation causes nucleation of perovskite precursors before confinement by the PVP matrix, leading to large-sized PeNCs. Vacuum drying of perovskite ink at low temperature can effectively confine the PeNCs within the PVP matrix, leading to a uniform and smooth dot profile. On the other hand, the high temperature drying process induces the coffee ring effect due to the reduced droplet viscosity and increased outward capillary flow.

As part of the postprocess, not only the substrate temperature [158] but drying condition also determines the quality of perovskite crystallization. Wang et al. compared the qualities of quasi-2D perovskite films formed under different drying conditions [22]. Under ambient conditions, high humidity and oxygen during the drying process resulted in films with a 3D structure and porous morphology, characterized by numerous cracks. Crystallization in a vacuum environment accelerated solvent evaporation, resulting in a high density of nucleation sites and shorter crystallization time. This led to films with reduced grain size, decreased surface roughness, and increased low-dimensional components. Rapid solvent evaporation in a vacuum also shortened the outward capillary flow time and facilitated the formation of uniform films without the coffee ring effect, as shown in Fig. 6d. Using inkjet printing, red, green, and blue matrices with 120 PPI resolution were fabricated, achieving maximum luminance and maximum EQE of 956 cd m^−2^ and 3.4% for green PeLEDs (Fig. 6e, f).

All-inkjet-printed PeLEDs can be successfully fabricated on elastic polymer substrate using successive inkjet printing of PEDOT:PSS/polyethylene oxide (PEO) as a transparent anode, CH_3_NH_3_PbBr_3_/PEO as an emitter, PEI as a buffer layer, and Ag nanowires (AgNWs) as a top cathode (Fig. 6g) [23]. A pinhole-free, intrinsically stretchable perovskite emitting layer was successfully fabricated through the optimization of the printing temperature. At 65 °C, by evaporating the residual solvent, uniform sized perovskite grains (5–8 μm) were crystallized within the PEO polymer matrix, while large dendritic grains with random sizes (20–40 μm) were generated at room temperature (Fig. 6h). To prevent corrosion of perovskite crystals caused by the solvent of the AgNW top electrode and to minimize the injection barrier, a branched PEI layer was incorporated as an interfacial buffer layer and electrode work function modifier. As a result, the all-inkjet-printed PeLED exhibited a maximum luminance of 10,227 cd m^−2^ and a current efficiency of 2.01 cd A^−1^. Additionally, the devise fabricated on polydimethylsiloxane (PDMS) substrate even shows good flexibility with bending radius of 2.5 mm (Fig. 6i).

Recently, the inkjet printing technique has been applied to create high-resolution patterns of single-crystal perovskites. Gu et al. reported single-crystal perovskite-embedded PDMS films (Fig. 6j) [159]. Interestingly, perovskite precursor droplets were generated through inkjet printing and then encapsulated within the liquid PDMS precursor. This space-confinement approach effectively slowed down the growth rate of perovskites, facilitating the formation of single-crystal perovskites (Fig. 6k). The study achieved high-resolution patterning with perovskite single crystals ranging in size from 6 to 17 μm (Fig. 6l), facilitating the realization of the complex images (Fig. 6m). Furthermore, the perovskite single crystals embedded in PDMS exhibited excellent mechanical flexibility and stability. Even after undergoing more than 1,000 bending cycles with the bending angle of 0–180°, the film maintained the original structure. In addition, the PL intensity showed no significant changes even after exposure to air for over 60 days.

### Electrohydrodynamic Jet (E-jet) Printing

**Fig. 7:**
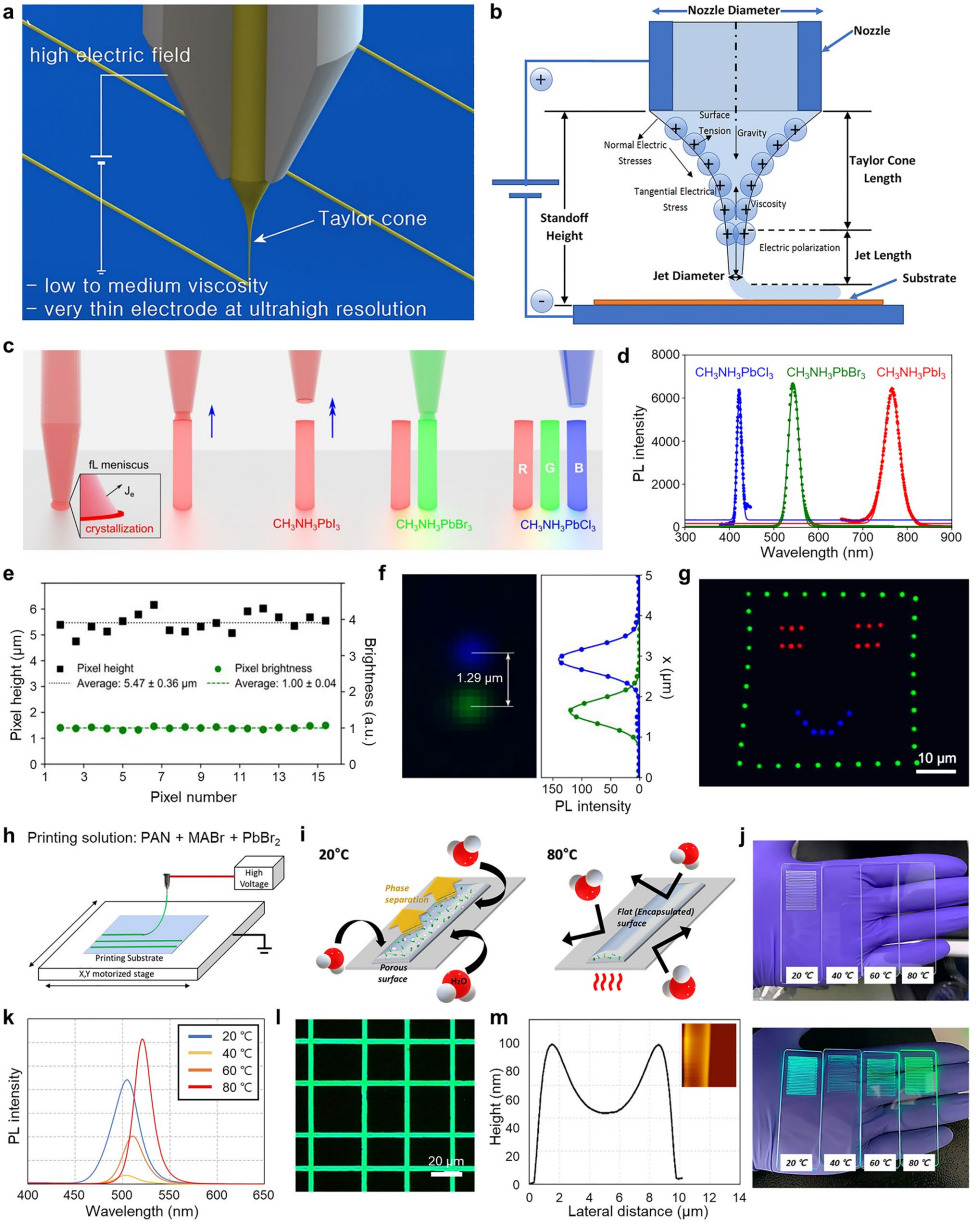
**a** Schematic illustration depicting the operational principle of E-jet printing. Reproduced with permission [26]. Copyright 2015, Springer Nature. **b** Schematic illustration showing the formation Taylor cone by applied voltage. Reproduced with permission [27]. Copyright 2021, Springer Nature. **c** Schematic illustration describing the formation process of 3D perovskite nanopixels through meniscus-guided crystallization. **d** PL spectra of red, green and blue 3D perovskite nanopixels. **e** The height and brightness distribution of 3D nanopixels. **f** High-resolution pixelated image with a gap of 1.29 μm and intensity profiling of multi-color pixels. **g** PL image of nanopixel patterns with RGB multi-color perovskite, showing a “Smile face”. Reproduced with permission [28]. Copyright 2021, American Chemical Society. **h** Schematic of the E-jet printing system with high viscosity printing solution. **i** The vapor-induced phase separation effect according to the processing temperature. **j** Photograph and **k** PL intensity of E-jet printed perovskite patterns according to the processing temperature. **l** PL image of single-color line patterns. Scale bar is 20 μm. **m** AFM image and line profile of the pattern. Reproduced with permission [29]. Copyright 2022, American Chemical Society

E-jet printing is a highly flexible patterning technique that offers several advantages, including contactless operation, and the absence of a photomask [160–162]. A precursor solution for perovskite formation is used as ink. One notable characteristic of E-jet printing, distinguishing it from conventional inkjet printing methods, is its utilization of an electric field established between the nozzle and substrate. This electric field generates electrohydrodynamic (EHD) forces that play a critical role in the process. These forces induce the formation of a stable cone-shaped liquid meniscus called the Taylor cone when a high electric potential is applied to the ink solution [26, 163]. Consequently, polarized droplets, much smaller than the diameter of the nozzle, can be precisely deposited onto a substrate (Fig. 7a), resulting in enhanced resolution compared to conventional inkjet techniques.

The process of creating the Taylor cone by E-jet printing and jetting droplets is affected by various experimental parameters (Fig. 7b) [27]. The size of droplets is mainly determined by variables such as viscosity, density, and surface tension of the ink materials, which ultimately determine the resolution of the final patterns. Additionally, the charges accumulated at the Taylor cone increase with the applied voltage, which can cause changes in the shape of the Taylor cone or the size of the droplets. The jetting speed (frequency), which is adjusted according to the applied voltage, should be carefully controlled, because it affects the dripping speed of the ink, which determines the desired patterning structure [164]. Furthermore, various parameters such as shape and size of nozzle and the pressure directly impact the E-jet printing process and the resulting patterning outcome.

Despite the complexity involved, E-jet printing offers promising advantages. Due to characteristics such as small droplet size and precise control of jetting speed, Wang et al. reported the fabrication of patterned perovskite films with the dot diameter of 1 μm for image sensor arrays [165]. Not only the high patterning resolution, but also it enables 3D nanoscale patterning, which is difficult to achieve with other printing technologies. Chen et al. proposed a strategy that utilizes solution-mediated evaporation-driven crystallization to create perovskite 3D nanopixels with desired height by balancing the precursor solution jetting speed and solvent evaporation rate (Fig. 7c) [28]. The crystallization of perovskite is discontinued by sudden rapid movements of the nozzle, which enables the reproducible formation of MAPbI_3_, MAPbBr_3_ and MAPbCl_3_ perovskite nanopixels with narrow emission peaks, constant height, and constant luminance (Fig. 7d, e). This approach does not involve the coffee ring effect, which is commonly observed in traditional inkjet methods and allows for the incorporation of the additional dimensional information through the formation of 3D structures. Furthermore, it was shown that the distance between pixels can be controlled down to ~ 1.29 µm, demonstrating the feasibility of high-resolution multi-color 3D patterning (Fig. 7f, g).

E-jet printing holds great promise for achieving high-resolution 2D and 3D patterning; however, it also presents challenges, particularly originating from the high viscosity of the ink solutions. In order to enhance the thickness uniformity, stability, and functionality of printed patterns, ink solutions usually contain polymer additives and have high viscosity. It is extremely difficult to generate small-sized droplets from highly viscous inks, which is essential for achieving high-resolution patterning. When the viscosity of the ink is high, the forces involved in droplet ejection may not be sufficient to overcome the cohesive forces within the ink [166]. Despite these challenges, advancements in E-jet techniques have shown the use of ink with high viscosity.

Kang et al. presented an E-jet printing process that incorporates polyacrylonitrile (PAN) into the perovskite precursor ink, resulting in improved flexibility, transparency, high water stability, and enhanced crystallinity (Fig. 7h) [29]. At lower temperatures, slow solvent evaporation leads to the formation of a porous structure through vapor-induced phase separation facilitated by water permeation (Fig. 7i) [167]. At an elevated temperature of 80 °C, the rapid evaporation of solvents suppresses the phase separation, resulting in the fabrication of perovskite films with dense and compact morphologies. These films exhibit high transparency and display narrow and bright PL characteristics (Fig. 7j, k). In addition, stripe patterns with the width of a few micrometers and the height of ~ 80 nm can be fabricated (Fig. 7l, m). This strategy addresses stability issues by demonstrating that perovskite films with resolutions of ~ 10 µm maintain PL intensity for up to 20 days in various polar solvents, including water.

The unique characteristics of the E-jet technique not only overcome challenges associated with inkjet printing but also enable the formation of transparent and flexible perovskite films with diverse structures, from 2D to 3D. These advantages hold significant potential for the future development of PeLEDs, and further advancements in the technology are expected.

### Thermal Evaporation

Thermal evaporation is a vapor deposition technique that is widely used for depositing thin films of various materials. It is a cost-effective and widely adopted method, particularly in large-scale patterning processes and industrial fabrication processes, such as the fabrication of OLEDs [168]. In this process, a solid material is typically heated within a vacuum chamber for vaporization. Subsequently, the vaporized atoms (or molecules) condense onto a growing substrate, forming a thin film. This technique, conducted under vacuum conditions, is particularly well-suited for perovskite materials, which are highly susceptible to degradation from exposure to water and oxygen [169]. The thermal evaporation process involves heating the perovskite material until it evaporates, followed by deposition of the resulting vapor onto a desired substrate [170–172].

Thermal evaporation of perovskites can be classified into three main methods depending on the number of precursor sources and the deposition steps: single-source evaporation, co-evaporation, and sequential evaporation. The single-source evaporation method employs a single precursor source by utilizing the characteristics of PeNCs decomposing at high temperatures [173]. Consequently, it can produce uniform and smooth films with minimal pinhole formation but has limitations in controlling the molar ratio of the precursor.

**Fig. 8:**
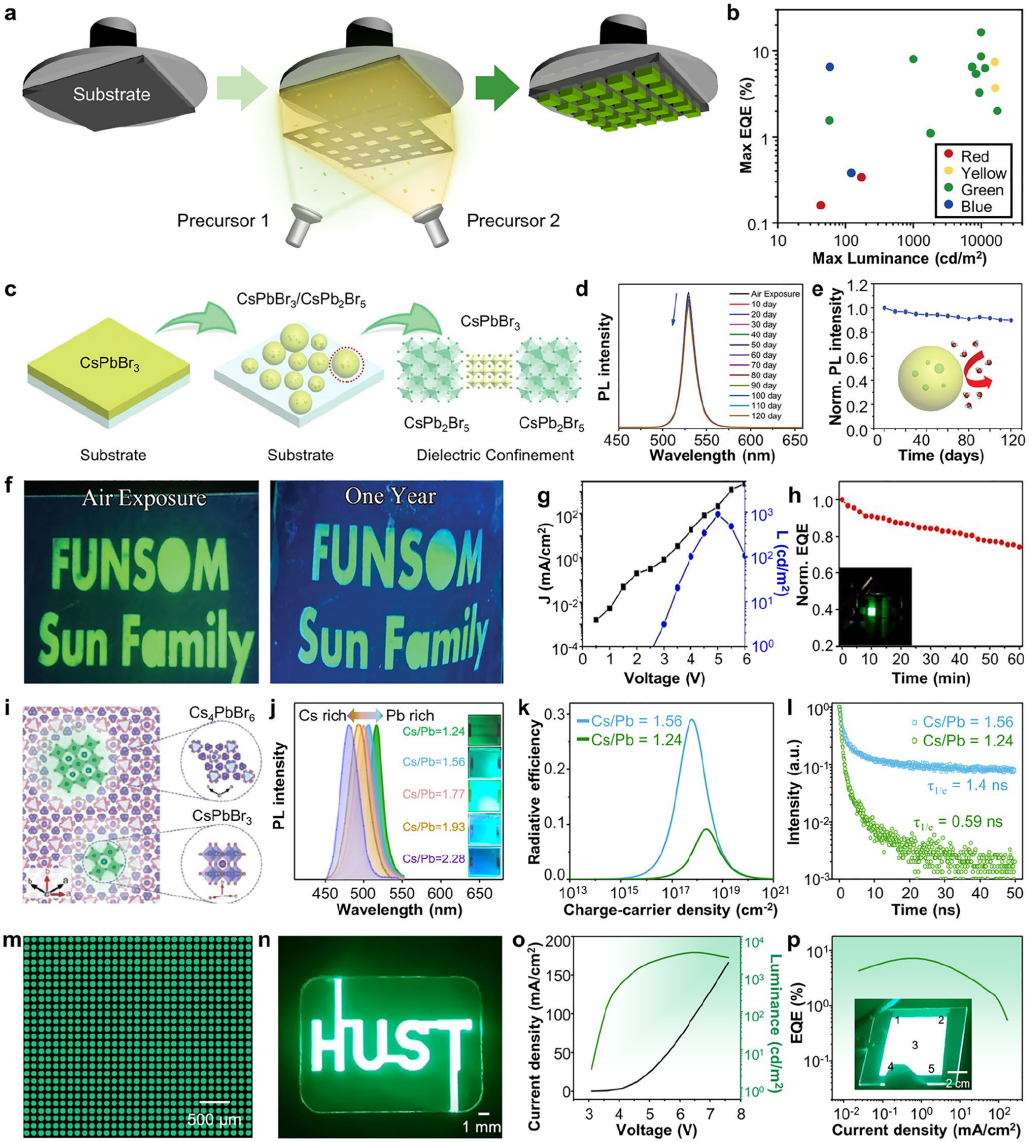
**a** Schematic illustration describing the co-evaporation process. **b** Max luminance and EQE chart for PeLEDs fabricated by the thermal evaporation of perovskite layers. **c** Schematic illustration showing the formation of matrix structure with CsPbBr_3_ embedded in CsPb_2_Br_5_. **d** Time-dependent PL spectra and **e** the PL peak intensity of perovskite films under air exposure (~ 60% humidity). **f** PL images of patterned perovskite films captured immediately after exposure to air conditions (left) and after one year in the ambient atmosphere (right). **g** *J–V–L* curve and **h** time-dependent EQE curve of PeLEDs employing the CsPbBr_3_/Cs_4_PbBr_6_ films. Reproduced with permission [32]. Copyright 2019, Wiley–VCH. **i** Schematic illustration showing the matrix structure with CsPbBr_3_ embedded in Cs_4_PbBr_6_. **j** PL intensity of perovskite films with various Cs/Pb ratios. **k** The simulated radiative efficiency versus charge carrier density and **l** time-resolved photoluminescence (TRPL) of perovskite films with Cs/Pb ratio of 1.56 and 1.24. **m** Fluorescence microscope image of the patterned perovskite film and **n** EL image of PeLEDs. **o** *J–V–L* curve and **p** EQE versus current density curve of large-area PeLEDs. Reproduced with permission [43]. Copyright 2021, Springer Nature

Co-evaporation, also known as multi-source evaporation, utilizes multiple precursors simultaneously. This enables the effective quantitative control of the molar ratio in the final products (Fig. 8a) [174]. However, controlling the simultaneous deposition of multiple precursors is challenging because the evaporation rate of precursors is determined by various physical properties including thermal conductivity and molecular weight as well as the external parameters such as the temperature and pressure. In contrast, sequential evaporation offers a comparatively straightforward solution by depositing the precursors layer by layer [175]. Nonetheless, the reaction between the solid-state layer, formed early in the process, and the vapor-state evaporated material may not proceed sufficiently due to the limited diffusion and contact area. In addition, several crucial parameters need to be considered in thermal evaporation, including the evaporation rate, molar ratio, type of substrate, and processing temperature.

Recent research indicated that PeLEDs fabricated using thermal evaporation technology have shown lower performance compared to those made by solution-based processes (Fig. 8b) [176, 177]. This can be attributed to the formation of relatively large crystal domains in perovskite films produced through thermal evaporation, leading to a smaller exciton binding energy and subsequently low efficiency of PeLEDs. Thus, there have been efforts on reducing the size of the crystal domains.

To enhance the performance of PeLEDs, surface passivation of PeNCs, which are sensitive to oxygen and water, has been suggested. Tan et al. reported the formation of matrix structure containing CsPbBr_3_ nanocrystals within the CsPb_2_Br_5_, showing enhanced moisture stability (Fig. 8c) [32]. Additional bromide ions, such as NaBr or LiBr, were introduced to create a high concentration of bromide environment, inducing the formation of CsPb_2_Br_5_. This approach resulted in the maintenance of a constant PL intensity even after 4 months of air exposure (Fig. 8d). Remarkably, even after 1 year, PL emission was maintained (Fig. 8e), demonstrating the high oxygen and water resistance of the CsPbBr_3_/CsPb_2_Br_5_ matrix structure. Another interesting effect observed was the reversible PL behavior induced by thermal effects. At high temperature conditions, nonradiative recombination become dominant, decreasing the optical performance. However, in the case of CsPbBr_3_/CsPb_2_Br_5_, the PL intensity could be recovered through a cooling process (Fig. 8f). This reversible process remained stable even after more than 300 cycles of heating and cooling between 0 °C and 100 °C. Furthermore, it was demonstrated that PL can be turned on and off through the thermal control using a heating circuit. However, when this perovskite film was applied to PeLEDs, it exhibited low luminance of 904 cd m^−2^ and an EQE of 0.3% (Fig. 8g), which can be attributed to the rough and non-uniform film structure. Also, EQE show time-dependently decrease under continuous current condition (Fig. 8h). Therefore, further optimization of the film structure is necessary to improve the device performance.

The presence of PbBr_6_^4−^ generated during the thermal evaporation process of CsPbBr_3_ perovskite can degrade the performance of PeLEDs by inducing nonradiative recombination through the dissociation of excitons [66]. Du et al. investigated methods to mitigate this issue to remove this octahedra structure [33, 169]. By controlling the molar ratio of Cs and Pb, CsPbBr_3/_Cs_4_PbBr_6_ composite film can be formed, in which Cs_4_PbBr_6_ can passivate CsPbBr_3_ layers (Fig. 8i). With increasing ratio of Cs/Pb, a high content of Cs_4_PbBr_6_ is observed. This indicates the formation of small-sized CsPbBr_3_ cores, as confirmed by the blue-shift in PL spectra (Fig. 8j). Cs_4_PbBr_6_ serves two important roles. Firstly, the longer exciton lifetime is observed for the samples with the Cs/Pb ratio of 1.56, suggesting that a higher production of Cs_4_PbBr_6_ results in a lower trap-assisted nonradiative recombination rate (Fig. 8k). Secondly, the stronger carrier confinement induced by Cs_4_PbBr_6_ enhances the probability of radiative recombination. As a result, the performance of PeLEDs was significantly improved due to the higher radiative efficiency achieved in the film with the Cs/Pb ratio of 1.56 (Fig. 8l). It was also demonstrated that large-area and high-resolution patterned perovskite film (pixel diameter =  ~ 100 μm) can be successfully fabricated and applied to PeLEDs (Fig. 8m, n). Large-area PeLEDs (40.2 cm^2^) exhibit the EQE of 7.1%, which is similar to the maximum EQE achieved for small devices (~ 8%). It can be achieved by employing the Li-doped NiO_x_ layer which acts as a HTL for enhancing the hole injection at room temperature. This suggests the feasibility of the large-scale production (Fig. 8o, p). This method has been further developed with the improved performances of PeLEDs. For example, Li et al. introduced a tri-source co-evaporation technique using additional ligands, which resulted in a high EQE of up to 16.4% [43].

## Patterning of Colloidal PeNCs

The terminology ‘patterning of colloidal PeNCs’ in this paper denotes printing techniques that utilize pre-synthesized PeNCs obtained through colloidal synthesis. The grain size of PeNCs is determined during the synthesis process, and their further growth is constrained by the ligands that cover their surfaces [178]. These uniform sized PeNCs effectively restricts the movement of excitons and provides smooth film formation, thereby enabling them to function as efficient emitters in PeLEDs. The functionality and processability of PeNCs can easily controlled by changing the solvent [179, 180] or surface ligands from long alkyl chains to short chains with desired functional groups [181, 182]. Interface engineering between PeNCs and CTLs also can enhance the patterning yield. In this chapter, we will discuss the recent developments in patterning technologies that utilize colloidal PeNCs and their applications in PeLEDs.

### Photolithography

**Fig. 9:**
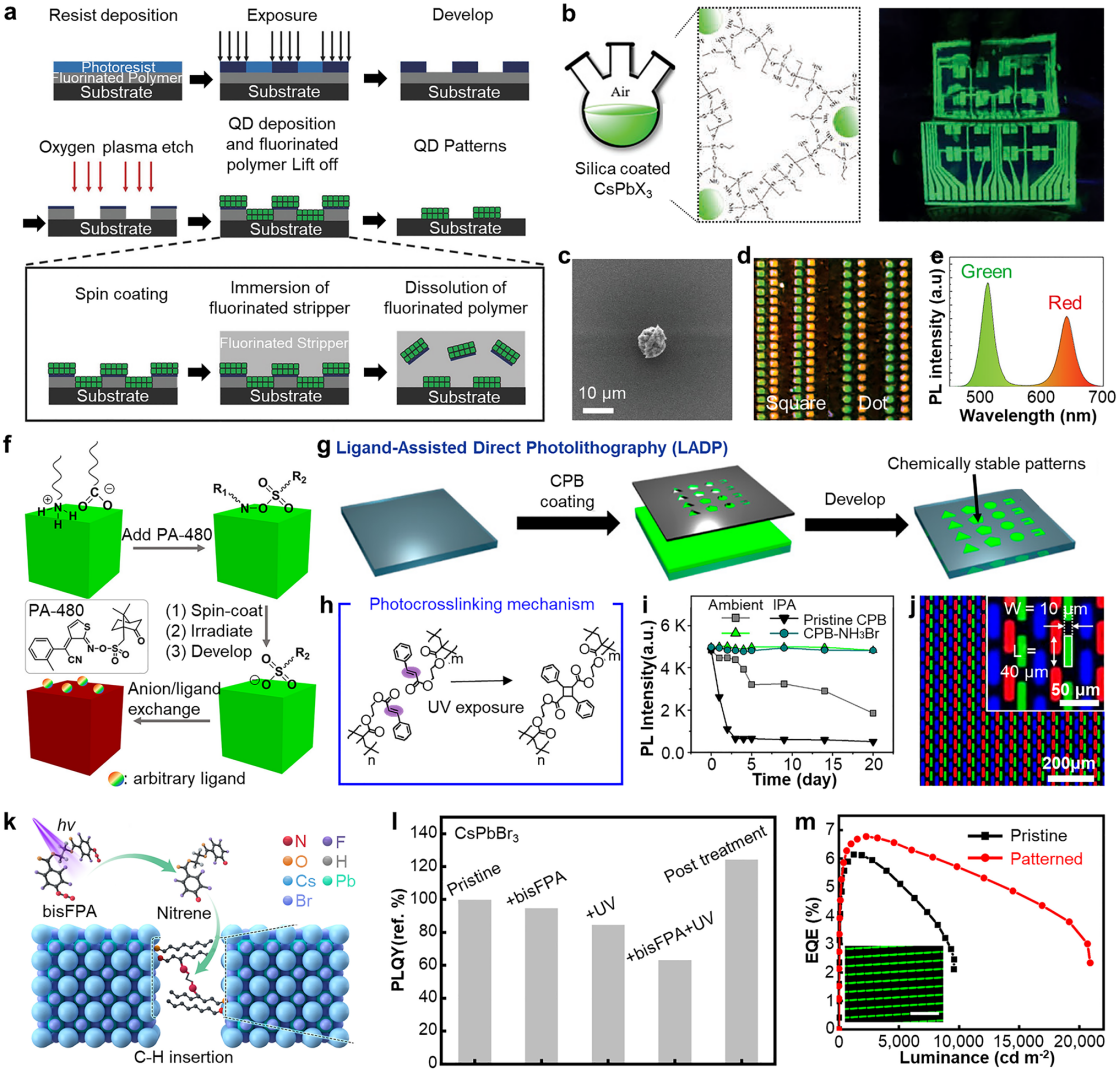
**a** Schematic illustration showing orthogonal photolithography process with fluorinated layer. Reproduced with permission [119]. Copyright 2018, Wiley–VCH. **b** Gel-type silica-coated perovskite precursor (left) and PL image of printed film on flexible PET substrate (right). **c** SEM image of silica-coated perovskite dot pattern with radius of 5 μm. **d** Periodic red/green square (left) and dot (right) patterns of silica-coated perovskites. **e** PL intensity of red and green patterns. Reproduced with permission [188]. Copyright 2020, Wiley–VCH. **f** Schematic illustration showing direct photolithography with photosensitive oxime sulfonate ester. Reproduced with permission [190]. Copyright 2021, American Chemical Society. **g** Schematic of ligand-assisted direct photolithography patterning. **h** Schematic illustration showing photocrosslinking mechanism using the cinnamoyl group on the ligand under UV exposure. **i** PL intensity versus time graph of pristine CPB and CPB-NH_3_Br under ambient, and IPA conditions. **j** PL image of RGB pixelated perovskite patterns by LADP patterning method. Reproduced with permission [191]. Copyright 2021, American Chemical Society. **k** Schematic of ligand crosslinking by bisazide under UV exposure. **l** Relative PLQY of CsPbBr_3_ films under different treatments in direct optical patterning processes with ligand crosslinker. **m** EQE performances of EL devices of pristine and patterned by DOPPLCER. The inset shows patterned EL device. Scale bar is 200 μm. Reproduced with permission [21]. Copyright 2022, The American Association for the Advancement of Science (AAAS)

The PeNCs can be patterned by photolithography in two ways: lift-off PeNC film using photopatternable polymer (i.e., photoresist) and direct patterning of photocurable PeNCs [39, 183, 184]. For the fabrication of printed PeLEDs, damage control onto the PeNCs (e.g., exposure to UV light and chemicals) during the photolithography is critical to preserve initial optical and electrical characteristic of PeNCs [185, 186]. Due to the inherent chemical vulnerability of PeNCs, the utilization of photolithography with commonly used photoresists and polar developer solutions cannot guarantee the optical properties of PeNCs. Lin et al. proposed an orthogonal photolithography technique using a fluorinated polymer and solvent for the fabrication of multi-colored complex CsPbBr_3_ PeNCs patterns (Fig. 9a) [119]. As a sacrificial layer, fluorinated polymer resist (OSCoR SL 1) was coated onto the substrate and patterned using conventional photolithography procedure. PeNCs were deposited onto the patterned fluorinated polymer and patterned by lift-off using fluorinated stripper. The usage of the fluorinated polymer addresses the challenges associated with polar-nonpolar solvent constraints, leading to the successful formation of high-definition PeNC patterns (~ 1,000 PPI).

The chemical stability of colloidal PeNCs can be tuned by surface modification of PeNCs. For example, core/shell type of PeNCs encapsulated by oxide materials can effectively enhance the stability of PeNCs during photolithography [187]. Oh et al. demonstrated highly stable gel-type silica-coated CsPbBr_3_ PeNCs using hydrolysis of (3-aminopropyl)triethoxysilane (Fig. 9b, left) [188]. The patterned silica-coated PeNCs, fabricated using photolithography and lift-off techniques, exhibited remarkable optical stability even in challenging environmental conditions, including high humidity (95%) and exposure to anti-solvents such as ethanol or methyl acetate (Fig. 9b, right). The feature size can be decreased to 5 $\upmu$m (Fig. 9c). Multi-colored patterns were demonstrated through the successive printing of red and green PeNCs (Fig. 9d, e). Encapsulating PeNCs with inorganic materials is an effective method to enhance their chemical stability. However, large bandgap of inorganic materials hinders the carrier injection into PeNCs, restricting the applications in PeLEDs.

Direct photopatterning of PeNCs offers a promising solution to mitigate the undesired exposure of PeNCs to solvents during the photolithography process, leading to improved optoelectronic characteristics in PeLEDs [189]. This technique involves the engineering of surface ligands on PeNCs to confer photocurable properties. Talapin group suggested direct photopatterning of PeNCs using a photosensitive oxime sulfonate ester (PA-480) as ligand of PeNCs (−C=N−OSOO−) [190]. As shown in Fig. 9f, irradiation of UV (405 nm) cleaves the N–O bond of the oxime sulfonate ester, resulting in the PeNCs becoming insoluble in the toluene developer. This solubility change enables the patterning of PeNCs. Moreover, the optical properties of the patterned PeNCs can be further enhanced through post-treatments involving anion and/or ligand exchange. Ko et al. exhibited photopatternable CsPbBr_3_ PeNCs by using a multifunctional polymer ligand of ammonium halide-terminated poly(2-cinnamoyloxyethyl methacrylate) (PCEMA–NH_3_X) (Fig. 9g) [191]. As shown in Fig. 9h, under UV irradiation (365 nm), the cinnamoyl group of PCEMA undergo crosslinking each other, resulting in the formation of stable PeNC patterns. These patterns exhibited excellent stability under ambient conditions and chemical exposure (Fig. 9i). The terminal group of PCEMA can be readily exchanged with ammonium halide, enabling the versatile multi-color patterning of PeNCs (subpixel size = 10 $\upmu$m × 40 $\upmu$m) through anion exchange (Fig. 9j).

Recently, patterned PeLEDs were reported by using direct photopatterning of CsPbBr_3_ PeNCs with bisazide ligand crosslinker [21]. Under UV irradiation (254 or 365 nm), bisazides generate reactive nitrene radicals, which exhibit a strong tendency to form covalent C–N bonds with the native ligands of PeNCs (e.g., oleylamine and oleic acid) through C–H insertion (Fig. 9k). The post-treatment involving a mixture of PbBr_2_/oleylamine/oleic acid in ethyl acetate leads to a remarkable increase in PLQY of PeNCs through the passivation of surface Br anions located on the PeNCs (Fig. 9l). Using this direct photopatterning procedure, pixelated PeLEDs were demonstrated with a maximum EQE of 6.8% and a max luminance of 20,350 cd m^−2^ (Fig. 9m). These results highlight the significant potential of photopatterned PeNCs for PeLED applications.

### Inkjet printing

Pre-synthesized PeNCs can serve as the ink for inkjet printing. The pre-synthesized colloidal PeNC ink simplifies the fabrication process by eliminating the post-annealing step, thereby reducing overall time requirement [156]. Additionally, the well-dispersed PeNC ink prevents undesired crystallization and growth of PeNCs during the printing process [192, 193]. Despite these benefits, long-term stability remains an issue for colloidal PeNC inks in effective inkjet printing of PeNCs [46, 194, 195]. Addressing the stability concerns of colloidal PeNC inks is crucial for ensuring their reliability and suitability for practical applications in inkjet-printed PeLEDs.

**Fig. 10:**
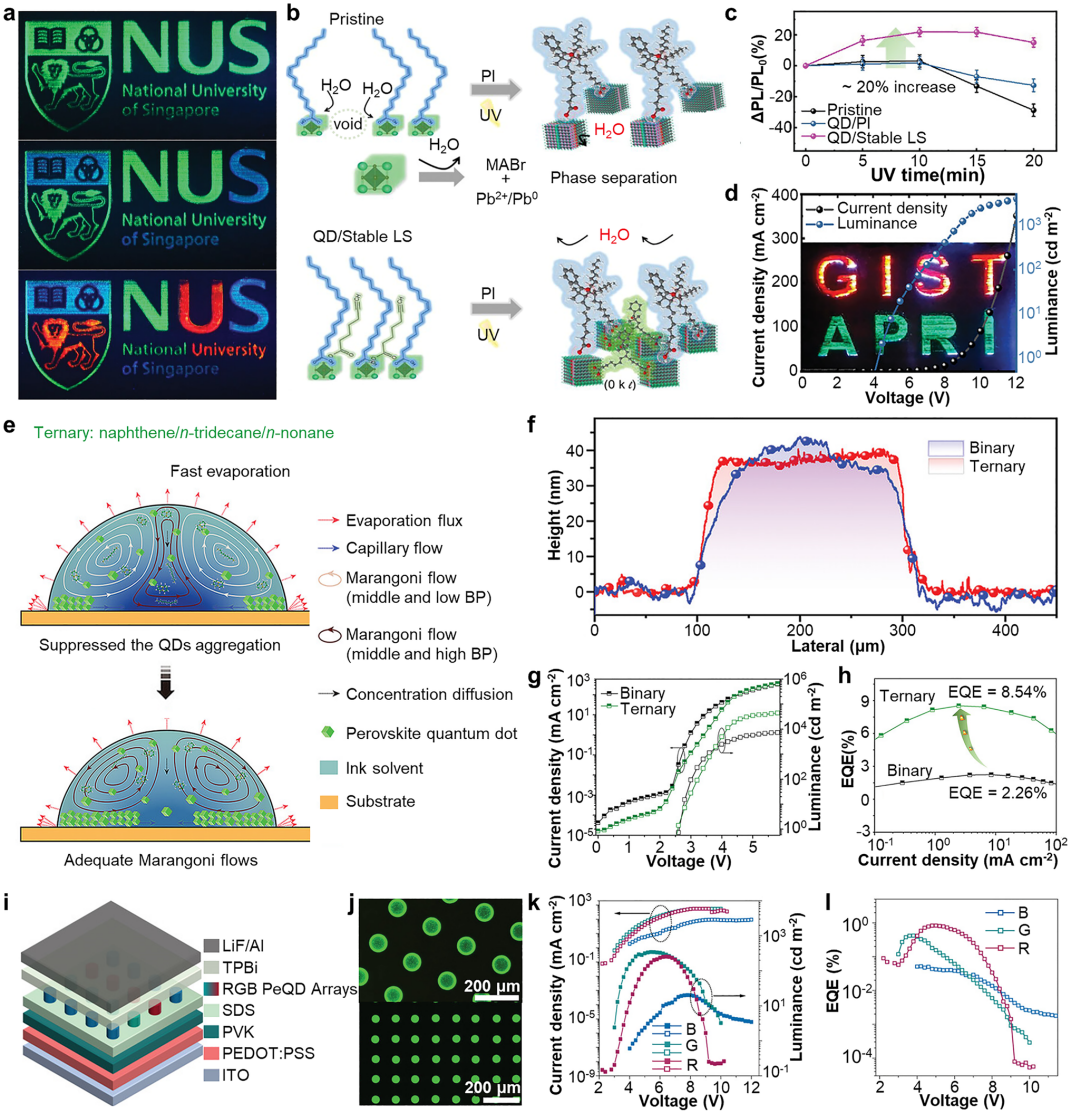
**a** PL image of sequential inkjet-printed RGB PeNC patterns through halide exchange using tert-butyl chloride and tert-butyl iodide. Reproduced with permission [196]. Copyright 2019, Wiley–VCH. **b** Illustration of pristine and UV-crosslinked PeNC degradation mechanisms under UV and ambient environment. **c** Time-dependent changes in relative PL intensity of pristine PeNCs, PeNCs with PI, and PeNCs with LS under UV exposure. **d** *J–V–L* curves of PeLEDs fabricated by inkjet printing of PeNCs. The inset shows inkjet-printed red and green EL device. Reproduced with permission [24]. Copyright 2021, Wiley–VCH. **e** Schematic illustration of droplet rheology with ternary solvent ink system. **f** Topographical profiles of inkjet-printed PeNC thin films obtained using a binary and ternary solvent ink system. **g** *J–V–L* curves and **h** EQE versus current density curves of inkjet-printed PeLEDs using the binary and ternary solvent ink system. Reproduced with permission [41]. Copyright 2022, Wiley–VCH. **i** Schematic of PeLED structure patterned by inkjet printing. **j** Fluorescence images of inkjet-printed PeNC arrays on PVK (top) and SDS (bottom). **k** *J–V–L* curves and **l** *EQE–V* curves of inkjet-printed RGB PeLEDs. Reproduced with permission [25]. Copyright 2022, Wiley–VCH

Wong et al. demonstrated the creation of inkjet-printed RGB PeNC patterns through the photoactivated halide exchange of PeNCs with molecular haloalkanes [196]. The halogen atom within the haloalkane interacts with vacancies on the PeNC surface, forming lead-halogen bonds. Simultaneously, the carbon radicals from the haloalkane make bonding with neighboring halogens on the PeNC surface. The activation energy and reaction rate of this process depend on the breaking of the carbon-halogen bond and stabilization of the carbon radical, which can be enhanced by photon energy. The micrometer-scale RBG PeNC patterns were achieved by successive inkjet printing process (Fig. 10a). In this process, tertiary haloalkanes were utilized as color conversion inks and sequentially printed onto green PeNC patterns. The specific choice of haloalkanes allowed for the efficient conversion of the green emission of the PeNCs into red and blue emissions, resulting in the desired RGB color output.

To enhance the stability of colloidal PeNC ink, Lee et al. proposed the utilization of stable ligand system composed of PeNCs, crosslinkable ligands (alkynoic acid), a photoactive agent, and a suitable solvent [24]. The key component of this system is the photocrosslinkable ligand, which was designed with two functional groups: a carboxyl group (−COOH) for interaction with PeNCs and alkyne group (C≡C) for UV-mediated chemical reaction. The short-chain alkynoic acid effectively fills the voids on the surface of PeNCs through rapid ligand exchange with the pristine surface ligands such as OA. Under UV exposure, the alkynoic acids undergo alkyne-to-alkyne reactions, forming covalent bonds between neighboring PeNCs (Fig. 10b). The crosslinked PeNCs restricted ligand dissociation and provided effective passivation of PeNCs against ambient environments and UV (Fig. 10c). By employing this stable ligand system as a colloidal ink, inkjet-printed PeLEDs were demonstrated with a maximum luminance of 2,800 cd m^−2^ for green emission (Fig. 10d).

To suppress the coffee ring effect, ternary solvent ink was proposed for the stable and efficient inkjet printing of CsPbX_3_ PeNCs [41]. The universal ternary solvent ink formulation consists of naphthene as the primary solvent, n-tridecane for viscosity control, and n-nonane for gradient volatilization. This formulation is designed to achieve favorable film morphology, precise viscosity adjustment, and controlled evaporation rate during the printing process. By prolonging the Marangoni flow through the gradual volatilization of n-nonane, the coffee ring effect was effectively suppressed, as shown in Fig. 10e. This ternary solvent ink exhibited exceptional stability, maintaining well-dispersed PeNCs for over 30 days. Remarkably, compared to binary solvent inks, the ternary solvent ink printed much smooth and uniform thin film (thickness ~ 50 nm), suitable for the emission layer of PeLEDs (Fig. 10f). By using this PeNC ink, inkjet-printed PeLEDs were manufactured with a maximum EQE of 8.54% and a maximum luminance of 43,883 cd m^−2^, while effectively minimizing leakage current (Fig. 10g, h).

Another promising approach to mitigating the coffee ring effect is interface engineering between the PeNC ink and target substrate [197]. As the poor wetting interface layer, sodium dodecyl sulfate (SDS) was introduced on the HTL of PeLEDs (Fig. 10i) [25]. Direct inkjet printing of cyclohexylbenzene based PeNC ink on PVK (HTL) resulted in uneven morphology of large-sized pixels (~ 80 mm) (Fig. 10j, top). However, the introduction of an SDS layer effectively reduced the droplet–substrate wettability and increased the contact angle, thereby eliminating the coffee ring effect and reducing the pixel size (~ 45 μm) (Fig. 10j, bottom). After printing of PeNC ink, plasma etching was performed to mitigate excessive recombination in the CTL within PeLEDs. The inkjet-printed RGB micro-PeLED pixels were demonstrated with maximum luminance of 272.2, 379.2, and 22.8 cd m^−2^ and maximum EQE of 0.832, 0.419, and 0.052% for red, green, and blue PeLEDs, respectively (Fig. 10k, l).

### Transfer Printing

**Fig. 11:**
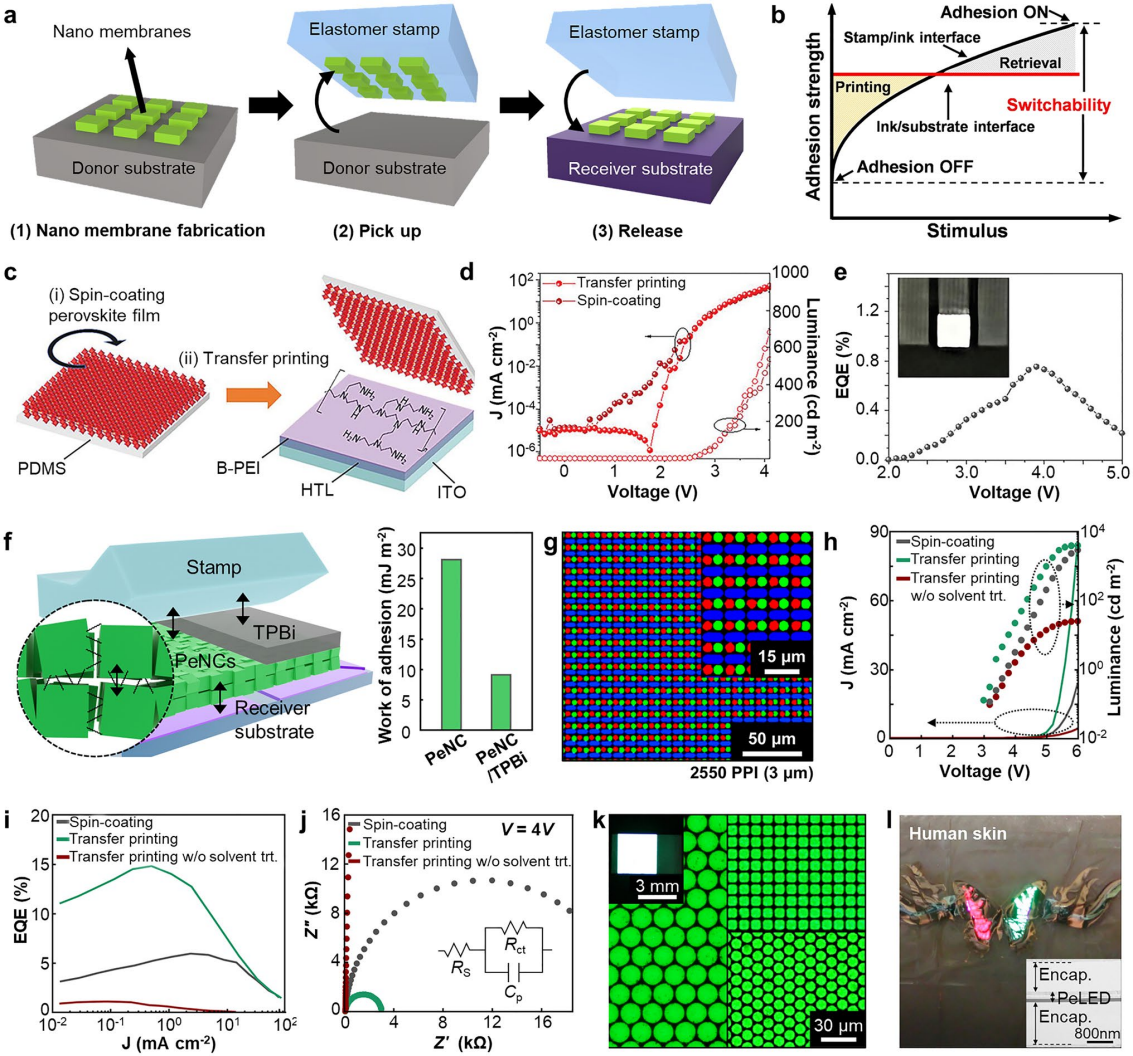
**a** Schematic illustration showing transfer printing process. **b** Diagram of adhesion versus stimulus considering the two interfaces in stamp/ink/substrate system. Reproduced with permission [204]. Copyright 2018, Springer Nature. **c** Schematic of mass transfer printing method. **d** *J–V–L* curves of red PeLEDs fabricated by transfer printing and spin-coating. **e** EQE curve of white PeLED. The inset show photograph of white PeLED. Reproduced with permission [30]. Copyright 2022, Wiley–VCH. **f** Schematic of PeNCs/ETL double layer release process (left) and work of adhesion graph between PDMS stamp and PeNC and PeNC/TPBi double layer (right). **g** Fluorescence microscopic images displaying pixelated RGB PeNC patterns with a resolution of 2,550 PPI. **h** *J–V–L* curves, **i** EQE versus current density curves, and **j** Electrochemical impedance analysis results of PeLEDs fabricated by spin-coating, transfer printing, transfer printing without solvent treatment of PeNCs. **k** Optical microscope images illustrating the EL emisstion from green PeLEDs using high-resolution transfer printing. The inset shows the photograph of green PeLED patterned by transfer printing. **l** Photograph of skin-attachable ultrathin multi-color PeLED. The inset shows a cross-sectional TEM image of a transfer printed PeLED with a total thickness of ~ 2.6 μm. Reproduced with permission [31]. Copyright 2022, American Association for the Advancement of Science (AAAS)

Dry transfer printing using viscoelastic PDMS stamp offers significant promise for achieving high-definition patterning of perovskite NCs, particularly for applications in EL devices [198, 199]. This method offers several advantages, including the absence of additional organic additives and the avoidance of solvent exposure during the patterning process [200, 201]. Typically, transfer printing involves two steps: rapid pick-up process from the donor substrate having low surface energy and release process to the target substrate using viscoelastic stamp (Fig. 11a) [202, 203]. In pick-up process, the separation energy between PeNCs/donor substrate (G^donor/PeNCs^) should be less than the energy between PeNCs/stamp (G^PeNCs/stamp^), while the separation energy between stamp/PeNCs (G^PeNCs/stamp^) should be less than energy between PeNCs/receiver substrate (G^PeNCs/receiver^) in release process (Fig. 11b) [204]. This ensures successful film transfer without damage or disruption. In contrast to the transfer printing of conventional luminescent nanoparticles (i.e., CdSe, InP, etc.) [205–207], transfer printing of PeNCs is still in the early stages of research. This is primarily due to the weak interaction between PeNCs, which leads to the formation of internal cracks within the film during the transfer printing process. Consequently, these cracks result in a reduced transfer yield of PeNCs.

To prevent the internal cracking of PeNC film during the transfer printing, Li et al*.* proposed transfer printing of PeNCs by directly spin-coating PeNCs onto the surface of PDMS stamp (Fig. 11c) [30]. The slow evaporation of PeNC solution on the stamp enables the large-area transfer printing (several centimeter scale) with low releasing pressure. Ultrathin branched-polyethyleneimine (B-PEI) layer having amine functional group is introduced as interfacial chemical bonding layer to enhance the adhesion and electrical contact between PeNC film and HTL. The transfer printed PeNC film achieves a monochromic pattern resolution of 1,270 PPI and demonstrated similar optical, mechanical, and electrical characteristics to the spin-coated counterpart. Thus, PeLEDs fabricated using transfer printed PeNC films exhibit a red emission with an EQE of 10.5%, comparable to PeLEDs employing spin-coated one (Fig. 11d). White PeLED is also demonstrated by multiple intaglio transfer patterning of red and sky-blue PeNC films, showing 80 cd m^−2^ of max luminance and 0.75% of max EQE (Fig. 11e).

Recently, double layer transfer printing of PeNCs/ETL is introduced to address the issue of internal cracking of the PeNCs film during the dry transfer printing [31]. The weak adhesion between the PDMS stamp and ETL layer facilitates the easy delamination of the PeNCs/ETL double layer from the stamp, without causing any damage to the PeNCs film (Fig. 11f). This double layer transfer printing enables the creation of high-definition RGB pixelated PeNC patterns with 2,550 PPI (Fig. 11g) and monochromic line patterns with 600 nm widths with almost 100% transfer yield. The PeLEDs with transfer printed PeNCs shows outstanding EL characteristics compared to the PeLEDs with spin-coated counterpart (Fig. 11h, i). Through the solvent treatment during the transfer printing procedure, the internal resistance is significantly reduced by control the interface between the HTL and PeNCs, resulting in enhanced carrier injection into PeNCs (Fig. 11j). High-definition green PeLEDs with 2,550 PPI (Fig. 11k) and ultrathin multi-color PeLEDs (Fig. 11l) are demonstrated using double layer transfer printing, showing the potential for the wearable displays.

## Conclusion and outlook

In this review, we have provided a comprehensive overview on recent advancements in patterning strategies for PeLEDs. In the case of in-situ crystallization patterning, the primary focus lies on patterning the precursor of MHPs. Because MHPs form during or subsequent to distinct stages of the patterning process, MHPs are less affected by various potential damages induced by solvents, light irradiation, and thermal influences during the patterning process. However, owing to their relatively larger and random distributed crystal size, which make less effective on the confinement of excitons and charges compared to the PeNCs [208], one of the solutions for this challenge is the realization of single-crystal MHP patterning. Single-crystal MHP films offer several advantages over polycrystalline counterparts, including higher carrier mobility [133, 159, 209] and an extended carrier diffusion length [207, 210–214]. Moreover, the in situ growth method holds the potential to further enhance device performance by eliminating unnecessary peeling processes which can possibly cause damage [215–218]. Nanocrystal patterning allows for precise control over the properties of the emissive layer, because the synthesis process, which is separated from the patterning process, can be finely controllable. For example, colloidal synthesis techniques can yield PeNCs with a uniform size distribution (standard size deviation < 1–2 nm) [31, 73], which is not achievable through the in situ crystallization patterning. Moreover, various post-synthesis surface treatment processes can be applied for PeNCs prior to the patterning process, playing a pivotal role in improving the electrical and optical properties of PeNCs [219] as well as the patterning yield [205, 220]. As we discussed, PeLEDs have garnered attention due to their wide color tunability, high carrier mobility, and high absorption coefficient. However, there are still several unresolved issues in the development of full-color PeLEDs, such as short lifetime under applied voltage, toxicity of Pb, circuit integration, and deformable displays.

The poor lifetime issue of PeLEDs under external bias must be ensured prior to commercializing the PeLEDs. The commercial OLEDs exhibit remarkable EQE (> 30%, approaching their theoretical limit) and lifetimes exceeding 100,000 h. PeLEDs also shows high luminous efficiency close to the theoretical limit, but most of their lifetime is still only a few hours [221, 222], which falls significantly short of meeting commercial requirements. The low stability is mainly caused by the ion migration of perovskites under external bias [223, 224]. To mitigate this challenge, core/shell type of heterogeneous structures are suggested. The core MHPs are encapsulated with wide bandgap materials to restrict the ion migration. For instance, Lee group suggested core/shell structured PeNCs with BPA shell [14]. The PeLEDs represent half-lifetime of 520 h at 1,000 cd m^−2^ (estimated half-lifetime > 30,000 h at 100 cd m^−2^), highlighting the potential of PeLEDs for commercialized displays. In this respect, various surface protection strategies have been recently suggested [225–229].

The stability issue caused by ion migration dominants in blue PeLEDs [230, 231]. The manufacturing process for blue PeNC film can be categorized into two directions: mixed halide perovskite and dimensional control for quantum confinement effects. Blue PeLEDs fabricated using mixed halide perovskites often suffer from a critical weakness in display applications, namely halide segregation during device operation [230, 232]. This phenomenon leads to an undesirable emission peak shift, which compromises the performance and stability of the devices. Recently, Jiang et al. demonstrated the use of monodispersed perovskite quantum dots by using ligands to induce steric hindrance [15]. This significantly improved efficiency and helped avoid halide segregation. However, the operational stability of these devices is still low, and further efforts are needed to achieve high efficiency and stable blue PeLEDs. The efficiency and stability of the devices can also be enhanced by effectively balancing the charge injection into PeNCs, preventing nonradiative Auger recombination and Joule heating. In addition, comprehensive understandings on their degradation mechanism would be highly demanding, as recently studied for semiconductor nanocrystals [233–235].

The toxicity of Pb, which poses significant environmental and health risks [236–239], is a hurdle for the widespread adoption of PeLEDs [240, 241]. Extensive research has been conducted to explore the substitution of Pb with low-toxicity metal cations, including Sn (II), Bi (III), Ge (II), Cu (I), Cu (II), Sb (III), Sn (IV), Ti (IV), and Ag (I) [242–245]. Particularly, elements in the same group such as Sn and Ge are favored element for lead-free perovskites. However, the thermodynamically favorable oxidation of Sn^2+^ and Ge^2+^ ions results in metallic properties and can lead to a reduction in device performance [246–248]. Furthermore, PeLEDs based on other elements have wide FWHM and stability issues, which are not appropriate for display applications [249, 250]. An effective approach to accelerate the discovery of promising candidates for lead-free PeLEDs involves the use of computational materials screening [251]. This methodology, widely employed in the solar cell industry, aids in identifying suitable materials with desired properties.

Integrating the PeLEDs with the backplane of the displays (i.e., transistor array) is essential to precisely control the intensity of individual RGB pixels. In conventional semiconductor manufacturing process, the Flip chip bonding is commonly used to interconnect with backplane and electronics [252]. During the connection of separately fabricated display units and circuit on a backplane through metal bumps of solder, Cu, and Au, additional annealing process and high pressure are required to ensure mechanical integrity and low contact resistance. As MHPs can be easily damaged under heat and pressure exposure, alternative approaches are required to integrate each unit. One promising approach is the direct fabrication of RGB PeLEDs on the backplane using MHP patterning strategies, such as inkjet printing [22], transfer printing [31], and thermal evaporation [43]. When integrating a PeLED array onto the TFT backplane or integrated circuits, it is crucial that the fabrication process of the PeLED array does not induce any damage to the underlying device circuits. For instance, monochromatic 1,080 × 2,400 active matrix PeLED array was successfully demonstrated by directly integrating top-emitting all-thermally evaporated PeLEDs on a 6.67-inch thin film transistor array [43].

With the recent advancement of electronic devices with various form factors [253, 254], the deformable displays have gained a great level of attention. The mechanical flexibility of LEDs is crucial for the fabrication of skin-attachable wearable displays [206] and the seamless integration with various wearable electronic devices [255, 256]. The mitigation of strain across each constituent—comprising electrodes, the CTLs, and the emissive layer—emerges as a pivotal requisite, particularly during diverse mechanical deformations including bending, folding, rolling, and stretching. One of the important strategies for the fabrication of deformable LEDs is structure engineering utilizing innovative designs such as serpentine, kirigami/origami, island, and ultrathin architectures [257–260]. Concurrently, an alternative method emerges through the employment of inherently stretchable materials [261, 262], propelling the development of stretchable CTLs, electrodes, emissive layers, and transistors to address these imperatives. While these strategies have been recently applied to create deformable PeLEDs [263, 264], there remains a compelling need for further improvements in terms of both EL properties and mechanical flexibility. By integrating these methodologies and processes, significant strides can be made in the realization of PeLED technologies for next-generation full-color displays in advanced electronic systems featuring diverse form factors [265–269].
